# Quantitative Phosphoproteomic and Physiological Analyses Provide Insights into the Formation of the Variegated Leaf in *Catalpa fargesii*

**DOI:** 10.3390/ijms20081895

**Published:** 2019-04-17

**Authors:** Nan Wang, Tianqing Zhu, Nan Lu, Zhi Wang, Guijuan Yang, Guanzheng Qu, Lisheng Kong, Shougong Zhang, Wenjun Ma, Junhui Wang

**Affiliations:** 1 State Key Laboratory of Tree Genetics and Breeding, Key Laboratory of Tree Breeding and Cultivation of State Forestry Administration, Research Institute of Forestry, Chinese Academy of Forestry, Beijing 100091, China; wwangnan@163.com (N.W.); tianqing.zhu@icloud.com (T.Z.); ln_890110@163.com (N.L.); wangzhi6666@126.com (Z.W.); yangguijuan123@163.com (G.Y.); sgzhang@caf.ac.cn (S.Z.); 2 State Key Laboratory of Tree Genetics and Breeding, Northeast Forestry University, Harbin 150000, China; quguanzheng@yahoo.com; 3 Department of Biology, Centre for Forest Biology, University of Victoria, Victoria, BC V8P 5C2, Canada; lkong@uvic.ca

**Keywords:** *Catalpa fargesii*, phosphoproteomics, variegated leaf, regulated mechanism

## Abstract

Variegated plants are valuable materials for investigating leaf color regulated mechanisms. To unveil the role of posttranslational modification in the variegated phenotype, we conducted global quantitative phosphoproteomic analysis on different leaf color sectors of *Maiyuanjinqiu* and the corresponding of *Catalpa fargesii* using Ti^4+^-IMAC phosphopeptide enrichment. A total of 3778 phosphorylated sites assigned to 1646 phosphoproteins were identified, and 3221 in 1434 proteins were quantified. Differential phosphoproteins (above 1.5 or below 1/1.5) in various leaf color sectors were selected for functional enrichment analyses. Gene ontology (GO) enrichment revealed that processes of photosynthesis, regulation of the generation of precursor metabolites, response to stress, homeostasis, amino acid metabolism, transport–related processes, and most of the energy metabolisms might contribute to leaf color. KEGG pathway enrichment analysis was performed based on differential phosphoproteins (DPs) in different organelles. The result showed that most enriched pathways were located in the chloroplasts and cytosol. The phosphorylation levels of glycometabolism enzymes might greatly affect leaf variegation. Measurements of fluorescence parameters and enzyme activities confirmed that protein phosphorylation could affect plant physiology by regulating enzyme activity. These results provide new clues for further study the formation mechanisms of naturally variegated phenotype.

## 1. Introduction

Variegated leaves usually consist of green and yellow/white sectors. Variegated species have outstanding application value in landscaping and landscape design. Moreover, they are ideal materials for studying the mechanism of leaf color formation. With advances in high-throughput technology, genomics, transcriptomics, and proteomics approaches have emerged in the study of variegated leaves [1,2,3,4]. It has been reported that the yellow sectors generally had decreased photosynthesis and increased oxidative stress to the green sectors by transcriptome and proteome profiling [1,5]. However, the correlation between mRNA and protein expression was low in the previous researches, and scholars postulated that posttranslational modifications (PTMs) might also play a pivotal role in variegation [6,7]. Protein phosphorylation is the most basic and most common mechanism for regulating and controlling protein activity and function, and it is closely related to many biological processes such as photosynthesis and oxidative stress [8]. We inferred that protein phosphorylation might have a great relationship with the phenotype. However, this speculation lacks detailed data. 

Protein phosphorylation could modulate the function of a particular protein, rather than achieving an overall effect by the overexpression/low expression or complete deletion of the gene encoding specific protein. Currently, the regulatory mechanism of photosynthesis proteins by phosphorylation modification is well established [9,10,11]. The well-known kinases STN7 and STN8 play important roles in optimizing photosystems under changing light and regulating PSII photo-damage repair [12,13]. In addition to phosphorylation studies of proteins localized in chloroplasts, an increasing number of phosphoproteins in other organelles have been researched [14]. Previous studies concluded that variegated plants were associated with the physiological phenomenon of state transition [15]. This phenomenon is often accompanied by phosphorylation of the PSII core subunits and the LHCII polypeptides [16]. It would be of interest to study the global levels of phosphorylation in the variegated leaves.

In recent years, the development of quantitative phosphoproteomics made it possible to detect phosphorylation sites on a global scale, which provided useful information for subsequent functional studies of the identified modification sites. At present, comparative phosphoproteomic analysis has been applied for the territory of plant growth, development and stress resistance [17,18,19]. However, the effect of phosphorylation on the natural variegated phenotype is still poorly understood. Comparative phosphoproteomic analysis will deepen our understanding of the effects of posttranslational modifications on leaf variegation.

*Catalpa fargesii* is a widely distributed native species in China. *Maiyuanjinqiu* is a novel variety of *C. fargesii* with natural variability, whose leaves display a typical variegated (yellow edge) phenotype (Identification code: 20150150). In our study, the green and yellow leaf sectors were sampled separately. The corresponding sectors of *C. fargesii* were used as controls. We performed comparative phosphoproteomic analyses using Ti^4+^-IMAC phosphopeptide enrichment technology based on TMT labeling. This enrichment technology has higher protein phosphorylation enrichment ability than TiO_2_ enrichment, conventional immobilized metal ion affinity chromatography (Fe^3+^-IMAC), and other approaches [20]. Integrative bioinformatics analyses were performed through screening differential phosphoproteins (DPs) among different color sectors. We also compared the fluorescence parameters and key enzyme activities in different leaf color sectors. This study not only helps to understand the role of protein phosphorylation in the variegated phenotype of *Maiyuanjinqiu*, but also provides a reliable reference for research on natural leaf color variation in other plants. 

## 2. Results

### 2.1. Phenotypic Character and Physiological Parameters in Maiyuanjinqiu and C. fargesii

**Figure 1:**
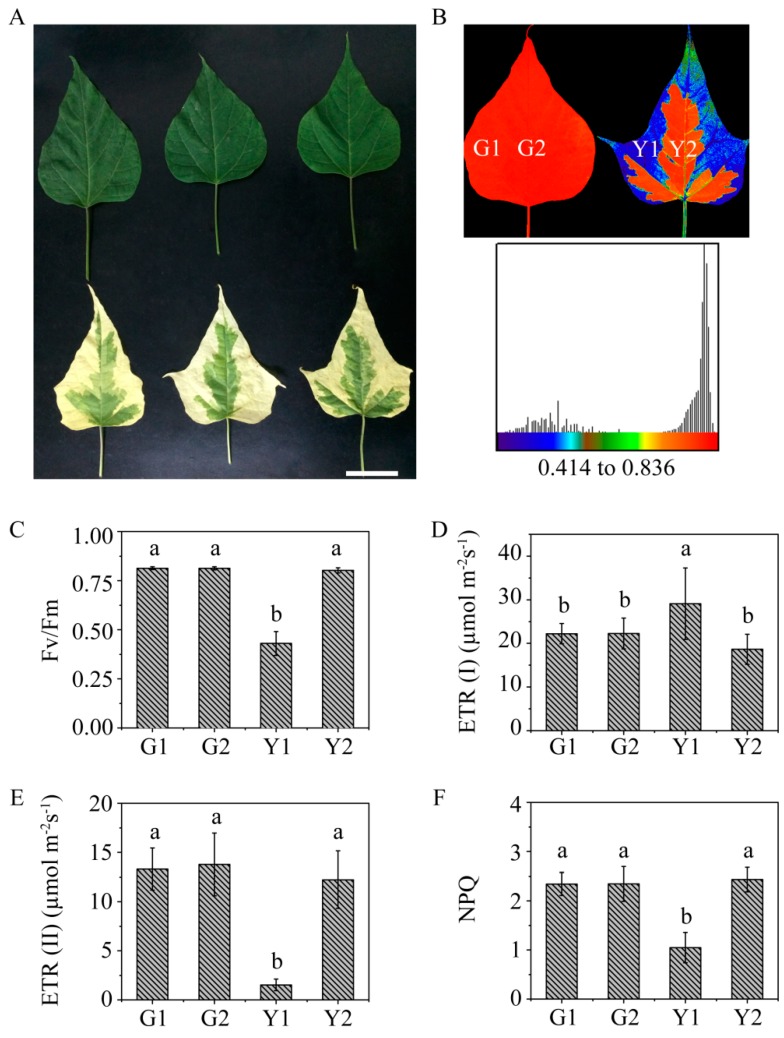
Phenotypic character and determination of photosynthetic fluorescence parameters. (**A**) Leaf phenotypes of *C. fargesii* (top) and *Maiyuanjinqiu* (down); the length of white bar represents 5 cm. (**B**) Fluorescence imaging of Fv/Fm; (**C**) The value of Fv/Fm; (**D**) The value of electron transport rate (ETR(I)); (**E**) The value of ETR(II); (**F**) The value of nonphotochemical quenching (NPQ) in G1, G2, Y1, and Y2. The photosynthetically active radiation was 129 µmol·m^−2^·s^−1^. Bars express the means ± SD (*n* = 18), and different letters indicate significant differences (*p* < 0.05, one-way ANOVA).

The leaves of *C. fargesii* were green throughout the whole growth period. *Maiyuanjinqiu* displayed a variegated leaf phenotype (Figure 1A). Photosynthetic fluorescence parameters were measured in *Maiyuanjingqiu* (Y1 and Y2) and the corresponding positions of *C. fargesii* (G1 and G2), respectively (Figure 1B). Fv/Fm represents the maximum photochemical quantum yield of PSII. The values of Fv/Fm were considerably lower in the yellow sectors than in the green sectors, whereas the values were similar in the Y2 and G2 sectors (Figure 1C). Compared with that in the green tissue, the electron transport rate of PSII (ETR(II)) was weakened in the yellow tissue, whereas the electron transport rate of PSI (ETR(I)) was enhanced under 129 µmol·m^−^2·s^−1^ in the yellow sectors (Figure 1D,E). These suggested that the energy balance of PSII and PSI was reequilibrated in the yellow sectors. The nonphotochemical quenching (NPQ) in Y1 was also significantly lower than that in the green sectors, which implied a deficiency in photoprotective ability in the yellow sectors (Figure 1F).

**Figure 2:**
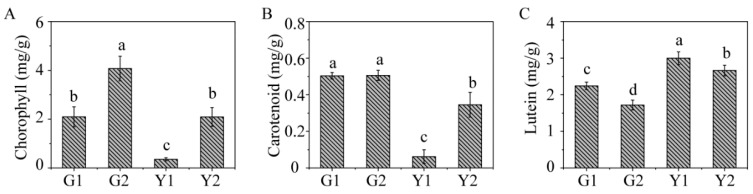
The measurement of pigment contents. (**A**) The level of chlorophyll; (**B**) The level of carotenoid; (**C**) The level of lutein. The values are shown as the means ± SD (*n* ≥ 4), a–c indicate that the differences are significant at *p* < 0.05 (one-way ANOVA).

The chlorophyll, carotenoid and lutein were examined in G1, G2, Y1, and Y2, respectively. The results showed that the total chlorophyll and carotenoids were much lower in *Maiyuanjinqiu* than in *C. fargesii* (Figure 2A,B). However, the level of lutein was higher in both the green and yellow sectors of *Maiyuanjinqiu* compared to the corresponding sectors of *C. fargesii* (Figure 2C). In addition, the chlorophyll and carotenoid contents were higher in Y2 than in Y1, but the level of lutein was lower in Y2. 

### 2.2. Analyses of Global Phosphorylated Proteins and Sites in Maiyuanjinqiu and C. fargesii

To assess the changes in protein phosphorylation on the variegated phenotype, we carried out a quantitative phosphoproteomics analysis in different leaf color sectors with three biological replicates. In total, 3778 phosphorylation sites in 1646 proteins were identified, among which 3221 sites in 1434 proteins were quantified. The number of total phosphopeptides was 2804 and the enrichment efficiency was 55.4%, respectively. First, the error rate of enriched phosphorylated peptides was counted. All peptide mass error rates were between −5 and 5 ppm, which suggested the high accuracy of the mass spectrometry data (Figure S1A). The number of modification sites for each phosphorylated protein in the phosphorylome was counted. Among which, approximately 54.9% proteins were singly phosphorylated. Phosphoproteins with two, three, four and greater than or equal to five sites comprised 21.4%, 9.19%, 6.34%, and 8.15% of the total (Figure S1B). The distribution of phosphorylated sites in our study was consistent with that in other plant studies [21]. In addition, the length distribution of more than 90% of peptides was between 7 and 18 (Figure S1C), which was consistent with the properties of tryptic peptides. Statistics on the phosphorylated amino acids revealed that the percentages of phosphorylation at serine, threonine and tyrosine residues were 78.3%, 15.3%, and 6.4%, respectively (Figure S1D).

### 2.3. Secondary Structure and Motif Analysis of Phosphoproteins

**Figure 3:**
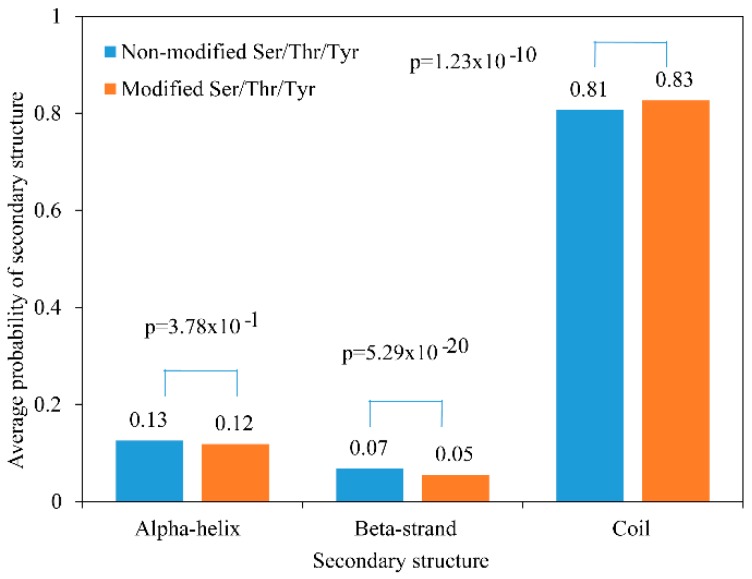
Probabilities of localization to different protein secondary structures.

To explore the relationship between phosphorylation and protein secondary structure, we performed a secondary structure analysis of all identified phosphorylated proteins. Approximately 83% of the phosphorylated proteins were in the disordered secondary structure region, while only 17% of the phosphorylated proteins were in the ordered secondary structure region (Figure 3). The modified and non-modified proteins were significantly different in the proportions of beta-strand and coil regions, but not of alpha-helix regions (Figure 3).

**Figure 4:**
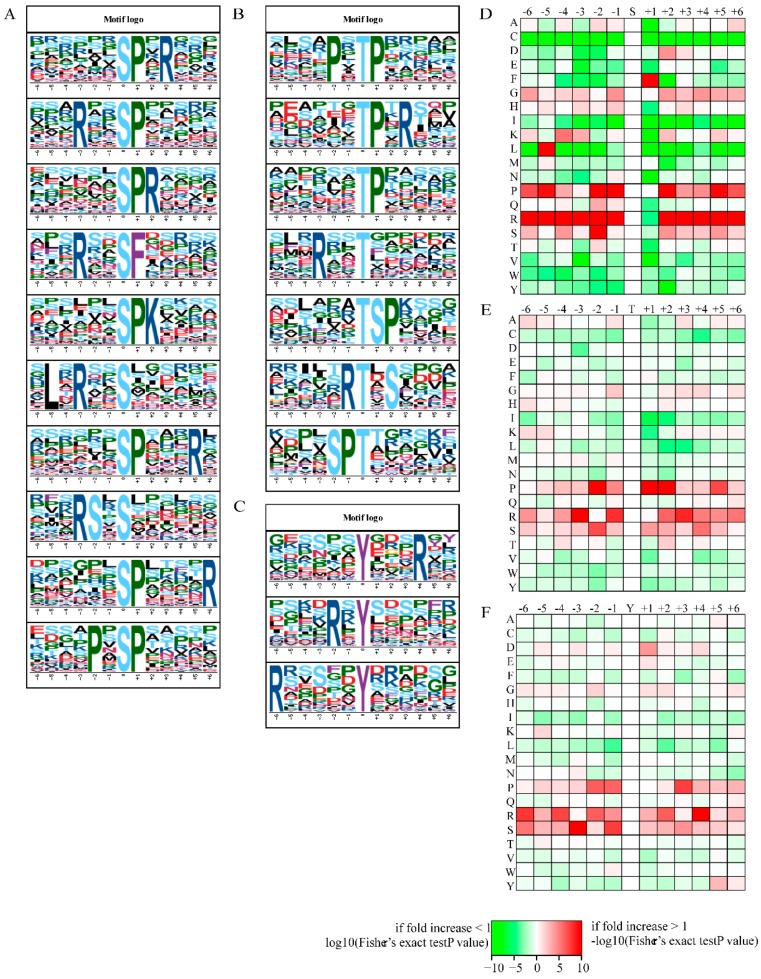
The motif analyses of modified sites. (**A**) Visualized motif analysis logos of serine (the top ten); (**B**) Visualized motif analysis logos of threonine; (**C**) visualized motif analysis logos of tyrosine; (**D**) conserved amino acid around the modified serine; (**E**) conserved amino acid around the modified threonine; (**F**) conserved amino acid around the modified tyrosine residues from −6 to +6 positions.

A motif analysis of the modified amino acid residues from −6 to 6 positions was performed. We identified 28 motifs at serine residues (Figure 4A), 9 motifs at threonine residues (Figure 4B) and 3 motifs at tyrosine residues (Figure 4C). The results showed that arginine (R) and proline (P) near the serine residue (S) were the most conserved amino acids upstream and downstream of modified serine sites (Figure 4D). R, P, and S were enriched around the threonine phosphorylated sites at the −3 to +3 positions (Figure 4E). The conserved amino acid R around the tyrosine modified site was mainly located at the −6, −2, and +4 positions (Figure 4F). These conserved amino acids near serine, threonine, and tyrosine might be functionally important for the occurrence of phosphorylation events. 

### 2.4. Identification of Differential Phosphoproteins (DPs)

**Figure 5:**
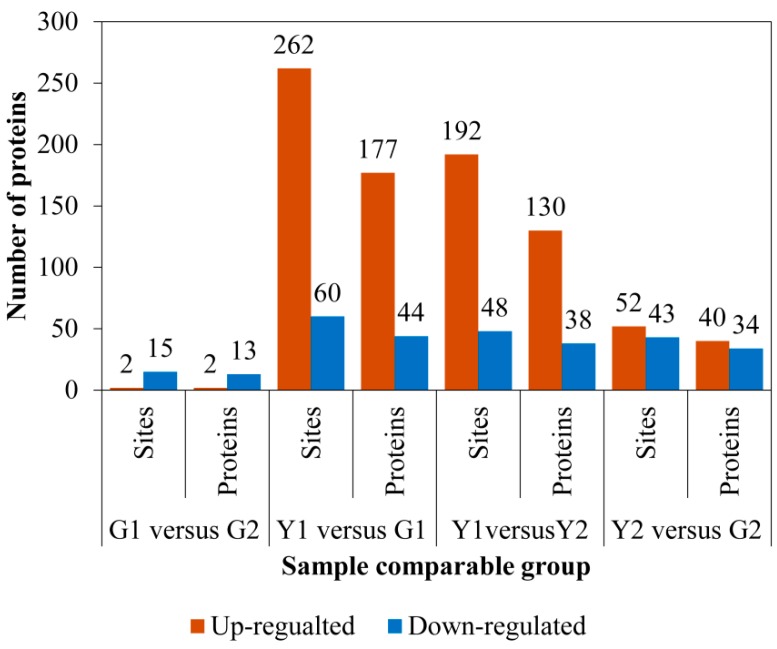
Numbers of phosphorylated proteins and sites in the G1 versus G2, Y1 versus G1, Y1 versus Y2, and Y2 versus G2 comparisons.

As shown in Figure 5, 221 DPs and 322 differential phosphorylation sites were identified in the Y1 versus G1 comparison, while 74 DPs and 95 differentially phosphorylated sites were identified in the Y2 versus G2 comparison. In the Y1 versus Y2 comparison, 168 differentially phosphorylated proteins and 204 differentially phosphorylated sites were identified. G1 versus G2 has only 15 differentially phosphorylated proteins and 17 phosphorylation sites, which are used to eliminate the positional effect between the Y1 and Y2 sectors. The upregulated DPs and differentially phosphorylated sites were significantly more frequent than the downregulated DPs and sites in the Y1 versus G1 and Y1 versus Y2 comparisons.

### 2.5. Gene Ontology (GO) Functional Classification, Subcellular Localization, and GO Enrichment Analysis

GO functional classifications were performed in the Y1 versus G1, Y2 versus G2, and Y1 versus Y2 comparisons. In terms of biological processes, the DPs of three comparisons were all enriched in the metabolic processes, cell processes, single organism processes, localization, biological regulation, stress response, etc with a slightly different scale (Figure S2). For the cellular component category, the DPs were mainly distributed among membranes, cells, macromolecular complexes and organelles (Figure S3). For the molecular function category, DPs were mainly associated with binding, catalysis, transport and structural molecular activity (Figure S4). In terms of subcellular localization, the DPs were mostly located in the chloroplast, nuclear, cytosol, mitochondria and plasma membrane (Figure S5).

**Figure 6:**
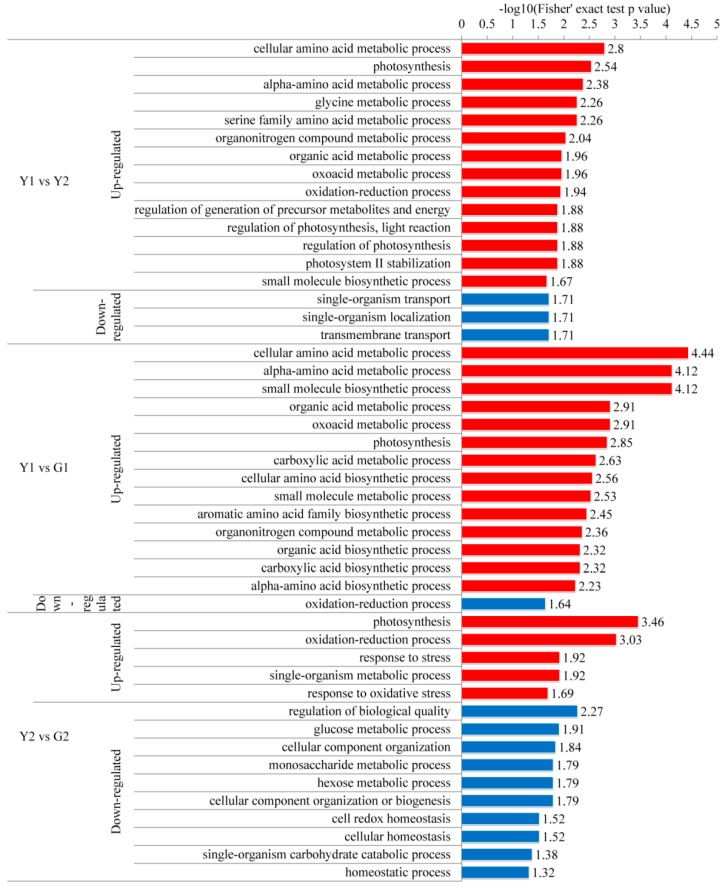
Gene ontology (GO) enrichment analysis of biological processes in the Y1 versus G1, Y1 versus Y2, and Y2 versus G2 comparisons. GO enrichment analysis of upregulated and downregulated proteins were separately.

GO enrichment analysis were performed based on the upregulated and downregulated levels of DPs in different comparisons. The GO terms of the Y1 versus G1 comparison were very similar to those in the Y1 versus Y2 comparison. In terms of biological processes, the processes of photosynthesis, amino acid metabolism, organic nitrogen complex metabolism, organic acid metabolism, oxoacid metabolism, redox process, and regulation of generation of precursor metabolites were upregulated in the yellow sectors, while processes associated with transport and localization were downregulated (Figure 6). In the Y2 versus G2 comparison, DPs involved in photosynthesis, oxidation–reduction process and response to stress were upregulated, while monosaccharides metabolic processes, glucose and hexose metabolic processes, single-organism carbohydrate catabolic processes and homeostatic processes were downregulated (Figure 6). For the cellular component category, the DPs in Y1 versus G1, Y1 versus Y2 and Y2 versus G2 shared a similar distribution, and were mostly located in the photosynthetic membrane, photosystem, thylakoid and thylakoid membrane (Figure S6). The differences are that non-membrane bounded organelles were downregulated in the Y1 versus G1 comparison, and cell and ribonucleoprotein complexes were downregulated in the Y2 versus G2 comparison. With respect to molecular function category, oxidoreductase activity and binding activity were upregulated, while transmembrane transport activity was downregulated in Y1 versus G1 and Y1 versus Y2 comparisons (Figure S7). In the Y2 versus G2 comparison, oxidoreductase activity, heme and tetrapyrrole binding activity, dioxygenase activity, and peroxidase activity were upregulated while carbon-carbon lyase activity and isomerase activity were downregulated (Figure S7). Taken together, the results showed that phosphorylation of proteins associated with photosynthesis, regulation of the generation of precursor metabolites, response to stress, homeostasis, amino acid metabolism, transport–related processes and most of the energy metabolic processes might contribute to leaf color. 

### 2.6. Domain Enrichment Analysis

Domain enrichment analysis showed that the phosphorylation levels of the ubiquitin-related domain, 1,3-beta-glucan synthase subunit, ClpP/crotonase-like, and glycoside hydrolase superfamily domain were significantly upregulated, while the chlorophyll a/b binding protein domain was downregulated in the Y1 versus G1 and Y1 versus Y2 comparisons (Figure S8). In addition, argonaute, linker domains that are required for RNA-mediated gene silencing, were upregulated in the yellow sectors. Similarly, the stress-related domain, including the thioredoxin-like fold and GroES-like domain, were also significantly enriched in the yellow sectors. Furthermore, the tetratricopeptide repeat-containing domain, nucleotide-diphospho-sugar transferases, ATPase, AAA-type, core and aldolase-type TIM barrel domain were enriched in the Y2 versus G2 comparison (Figure S8). The domain enrichment analysis further helps us to link functional domains and phenotypes.

### 2.7. KEGG Pathway of Differential Phosphoproteins in Different Sectors of Maiyuanjinqiu

**Figure 7:**
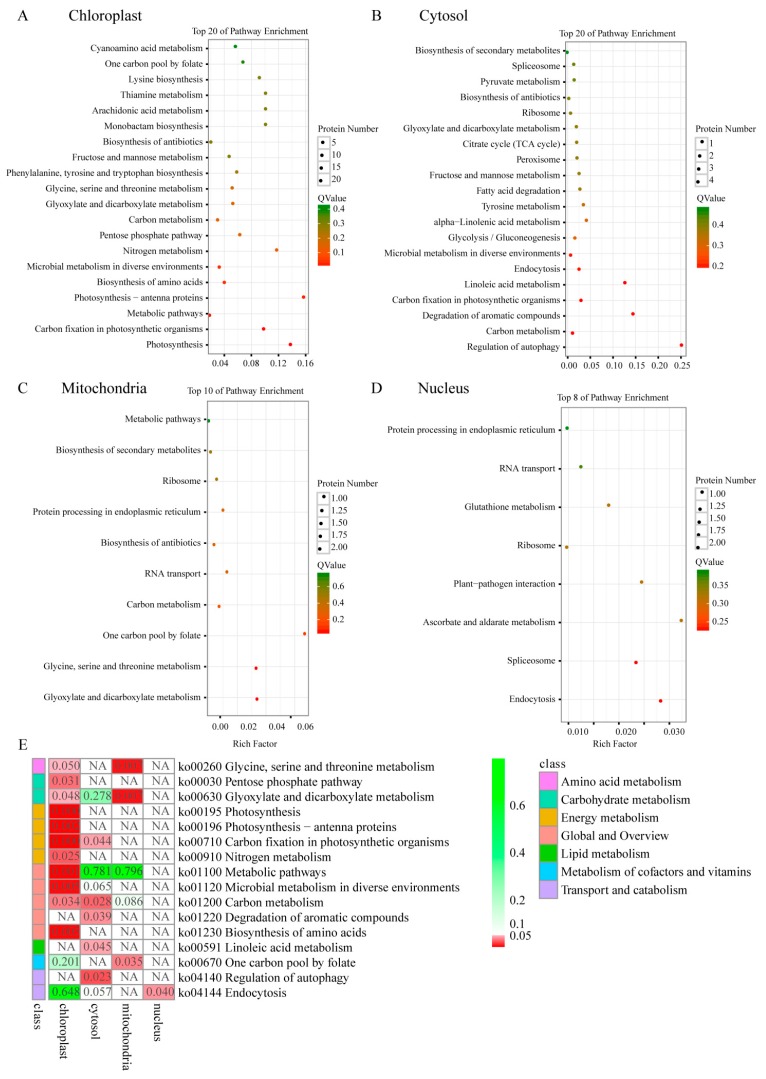
KEGG pathway enrichment of the specific differential phosphoproteins (DPs) between the green and yellow sectors based on the chloroplasts (**A**), cytosol (**B**), mitochondria (**C**), and nucleus (**D**), the common sites from the G1 versus G2 comparison were excluded. (**E**), the heat map of the qv value.

The specific phosphoproteins in the Y1 versus Y2 comparison were extracted. The common phosphoproteins identified in the G1 versus G2 comparison were removed. Further detailed KEGG pathway analysis of these specific DPs was performed based on the chloroplast, cytosol, nucleus and mitochondria. The DPs in chloroplasts were enriched in photosynthesis, photosynthetic antenna proteins, carbon fixation in photosynthetic organisms, nitrogen metabolism, carbon metabolism, pentose phosphate pathway, amino acid synthesis and microbial metabolism in diverse environments (Figure 7A). The DPs in the cytosol, DPs were mainly associated with carbon fixation in photosynthetic organisms, carbon metabolism, degradation of aromatic compounds, lipid metabolism and regulation of autophagy (Figure 7B). DPs on the mitochondria were mainly involved in amino acid metabolism, glyoxylate dicarboxylate metabolism and one-carbon pathway (Figure 7C). Although a considerable proportion of DPs were localized in the nucleus, only the endocytosis and spliceosome were significantly enriched in this subcellular compartment (Figure 7D). The DPs in the other components were mainly associated with transport and hydrolase activities. The results of KEGG pathway enrichment were consistent with those of the GO terms. By comparing the phosphorylation levels of different subcellular locations in the green and yellow sectors, we found that the central metabolic pathways were mainly enriched in the chloroplast and cytosol (Figure 7E). This result suggested that the phosphoproteins distributed in the chloroplasts and cytosol might play a key regulatory role in the formation of leaf variegation.

### 2.8. Protein–Protein Interaction (PPI) Network of Phosphoproteins 

Proteins in living organisms often do not function as single entities, but form a complex regulatory network to function together. The differentially phosphorylated proteins in the Y1 versus G1, Y1 versus Y2, and Y2 versus G2 comparisons were used to construct a PPI network. PPI network analysis of Y1 versus G1 and Y1 versus Y2 revealed that the differentially phosphorylated proteins between green and yellow sectors were mainly enriched in photosynthesis, ribosomes and a large number of metabolic pathways (Figure S9A,B). Phosphorylated proteins in the Y2 versus G2 comparison were mainly associated with photosynthesis, ribosomes and glycometabolism (Figure S9C). These phosphoproteins involved in photosynthesis, ribosome and energy metabolism might play an important part in the variegated phenotype.

### 2.9. The Key Enzyme Activities

**Figure 8:**
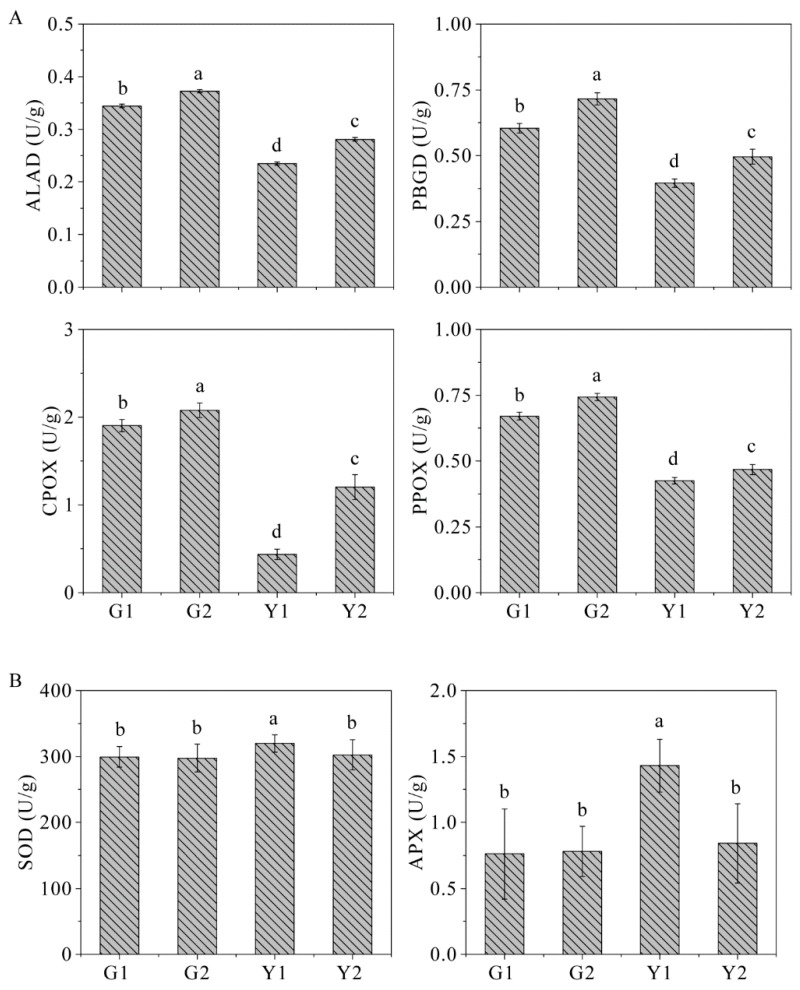
The key enzyme activities in chlorophyll synthesis and stress resistance. (**A**) The enzyme activities of ALA dehydrogenase (ALAD), porphobilinogen deaminase (PBGD), coproporphyrinogen III oxidase (CPOX), and protoporphyrinogen IX oxidase (PPOX); (**B**) the enzyme activities of superoxide dismutase (SOD) and ascorbate peroxidase (APX).

The activity of enzymes involved in chlorophyll synthesis, including ALA dehydrogenase (ALAD), porphobilinogen deaminase (PBGD), coproporphyrinogen III oxidase (CPOX), and protoporphyrinogen IX oxidase (PPOX), were further determined. The activities of these four enzymes were significantly lower in the yellow sectors than in the green sectors (Figure 8A). The activities of the antioxidant enzymes superoxide dismutase (SOD) and ascorbate peroxidase (APX) were also measured. The activities of SOD and APX were higher in the yellow leaf sectors than in the green sectors, which suggested that the ability to scavenge reactive oxygen was enhanced in the yellow sectors (Figure 8B). In addition, we enriched the phosphorylation of PBGD and APX in the yellow sectors, which suggested the phosphorylation of these enzymes might affect the processes of pigment biosynthesis and stress resistance by regulating the enzyme activities. 

## 3. Discussion

Previous researches have speculated that posttranslation modifications might play important role in the formation of variegation. However, the study of posttranslation modification is limited due to the scarcity of modified antibodies in plant kingdom. High- throughput phosphoproteomic can provide more effective information for us to understand the biological processes. In our study, we observed that the values of ETR (II) in the yellow leaves sectors were lower than those in the green sectors, while the ETR (I) values showed the opposite behavior (Figure 1D,E). These results suggested that the energy balance of PSI and PSII was redistributed in the yellow sectors, which was consistent with the fluorescence measurement results of the yellow cotyledons in the past [22]. This phenomenon attracted our attention to the differences in overall phosphorylation levels between the yellow and green sectors of *Maiyuanjinqiu*.

### 3.1. Global Analysis of Phosphorylome Profiling Associated with Leaf Color

In the Y1 versus G1 and Y1 versus Y2 comparisons, photosynthesis, carbohydrate/energy metabolism, protein metabolism, amino acid metabolic process, organonitrogen compound metabolic process, and the oxidation–reduction process were enriched based on GO enrichment (Figure 6). Our enrichment results shared similar pathways to those in the previous proteome of leaves from the chimera Hosta “gold standard” [6]. This result implied that protein phosphorylation might have a significant regulatory effect on protein expression. In the Y2 versus G2 comparison, the decreased phosphorylation levels of monosaccharides might contribute to the accumulation of increased levels of monosaccharides to respond and adapt to oxidative stress, achieving relative homeostasis in the green sectors of *Maiyuanjingqiu* (Figure 6). Combining domain enrichment analysis and PPI regulation network analysis, we concluded that photosynthesis and energy metabolism, protein homeostasis, and stress response were the key pathways associated with variegation. In the Y1 versus Y2 comparison (not include the common modified sites in the G1 versus G2 comparison), further KEGG pathway analysis was analyzed based on different organelle. The result showed that pigment biosynthesis, photosynthesis, energy metabolisms, stress response and defense, protein homeostasis, transcriptional regulation, and transport were most relevant to leaf color (Table A1).

### 3.2. DPs Involved in Pigment Biosynthesis and Photosynthesis

**Figure 9:**
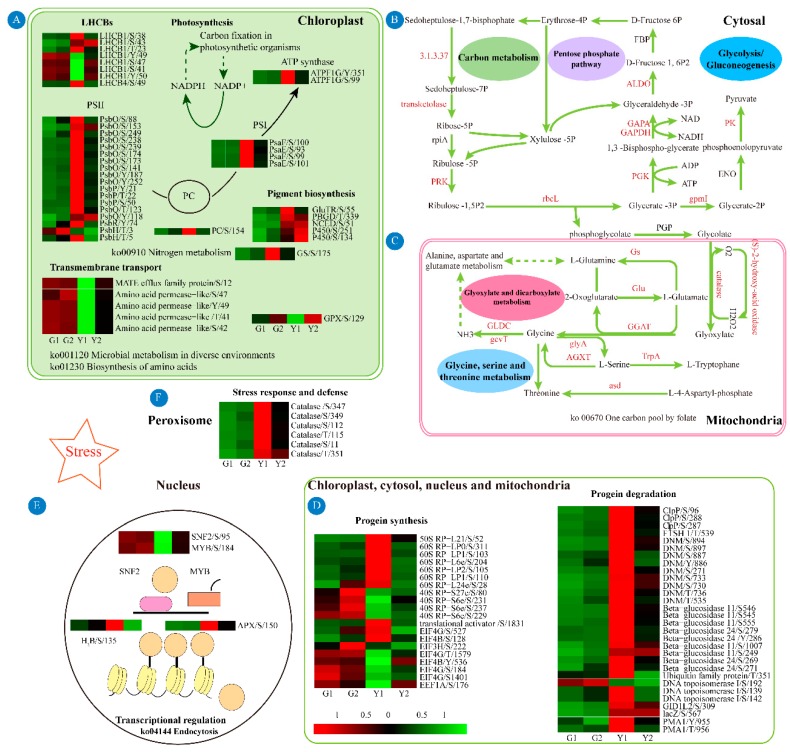
Schematic presentations of proteins involved in central metabolisms at phosphorylation level in green and yellow sectors. Differentially phosphorylated proteins are integrated according to subcellular organelles. (**A**) DPs localized on chloroplasts, including pathways such as photosynthesis, light-harvesting antennas and pigment biosynthesis. (**B**) DPs localized on the cytosal, including pathways such as glycolysis/gluconeogenesis, pentose phosphate pathway and carbon metabolism. The phosphorylated enzymes (upper right) are shown in red. The arrows represent chemical reactions. (**C**) DPs located on mitochondria, including glyoxylate and dicarboxylate metabolism, glycine, serine, and threonine metabolism. (**D**) DPs involved in protein synthesis and degradation is present in chloroplasts, cytosal, nucleus and mitochondria. (**E**) DPs localized on nucleus, including transcriptional regulators, such as transcription factors and histones. (**F**) DPs located on peroxisome, mainly stress- and defense-related proteins were presented. The expression pattern of phosphorylation levels are shown by heat maps in the G1, G2, Y1 and Y2 sectors. The phosphorylated proteins are upregulated or downregulated in red and green, respectively.

The phosphorylation levels of glutamyl-tRNA reductase (GluTR) and PBGD involved in chlorophyll biosynthesis were significantly higher in the yellow sectors than in the green sectors (Figure 9A). In contrast, the enzyme activities of ALAD, PBGD, CPOX, and PPOX involved in chlorophyll synthesis were lower in the yellow sectors (Figure 8A). These results implied that the phosphorylation of these two proteins might negatively regulate the enzyme activities, thereby affecting chlorophyll biosynthesis. Compared with the yellow sectors, the green sectors had higher phosphorylation levels of P450 family proteins (Figure 9A). P450 family proteins are involved in tetrapyrrole synthesis and catalyze the carotenoid synthesis process [23]. The globally higher phosphorylation levels of NCED in *Maiyuanjinqiu* might contribute to the biosynthesis of abscisic acid from carotenoids [24]. Taken together, the results indicated that phosphorylation levels of these DPs involved in pigment synthesis might be important targets for regulating leaf color. 

Light-harvesting antenna proteins are composed of pigment protein complexes. In our study, in addition to Thr 23, other phosphorylation sites of LHCB1 were all downregulated in the yellow sectors (Figure 9A). Studies have shown that the phosphorylation of LHCB1 is essential for optimizing the photosystem balance under changing environments [25,26]. The different phosphorylation sites of LHCB1 might play different roles in energy balance (Figure 9A). PsbO, PsbP, PsbQ, and PsbR are related to PSII stability [27]. The phosphorylation levels of these four proteins were upregulated in the yellow sectors, which might affect the distribution and rearrangement of protein complexes on the thylakoid membrane [28]. In addition, the phosphorylation levels of PSI proteins (PsaF and PsaE) were also upregulated in the yellow sectors compared to the levels in the green sectors (Figure 9A). The phosphorylation of these two proteins could affect their binding to plastocyanin (PC) and Fd and alter the distribution of LHCII to PSI [29]. The upregulated phosphorylation levels of photosynthesis-related proteins might be a regulatory adaptation mechanism for photosynthetic pigment deficiency in yellow leaves.

### 3.3. DPs Involved in Energy Metabolism

In addition to photosynthesis, energy metabolic processes were also significantly enriched based on GO enrichment and KEGG pathway analyses. The enriched pathways, such as glycolysis/gluconeogenesis, pentose phosphate pathway, carbon metabolism and nitrogen metabolism, were mainly located in the chloroplast and cytosol. In our study, a large portion of the enzymes involved in glycolysis/gluconeogenesis showed significant differences in phosphorylation levels between the green and yellow sectors (Figure 9B). For instance, fructose-bisphosphate aldolase (ALDO), EST C74302 (E30840) corresponds to a region of the predicted gene (GAPA), glyceraldehyde-3-phosphate dehydrogenase 2, cytosolic (GAPDH), putative 2, 3-bisphosphoglycerate-independent phosphoglycerate mutase (gpmI), 2-oxoglutarate dehydrogenase (OGDH), glucose-1-phosphate adenylyltransferase (glgC) and phosphoenolpyruvate carboxykinase (PGK) (Figure 9B). In addition, the pentose phosphate pathway is crucial to the maintenance of the redox state and plays a protective role in oxidative stress [30]. UDP-glucose 6-dehydrogenase (UGDH) catalyzes the first step of the pentose phosphate pathway. Transketolase (TK) synthesizes UDP-alpha-D-glucuronate from UDP-alpha-D-glucose, and acts as a stress sensor involved in the adaption process [31]. The expression of TK is related to the activity of the pentose phosphate pathway [32]. It has been reported that the balance between chloroplast and cytosol translation regulates the extent of *var2* variegation [33]. These phosphoproteins involved in glycometabolism on chloroplast and cytosol might be important for the formation of leaf variegation. Although studies have shown that posttranslational modifications of enzymes involved in glycometabolism can respond to plant growth, development, and stress tolerance [34,35], the regulation of leaf color by the phosphorylation of these enzymes was first reported recently. 

In addition to the large proportion of phosphorylated enzymes in glycometabolism, several enzymes involved in carbon fixation and carbon metabolism were also differential phosphorylated between the yellow and green sectors. For instance, phosphoribulokinase (PRK) is involved in the Calvin cycle, which is part of carbohydrate biosynthesis. Previous studies have suggested that posttranslational modifications of this protein might play a key function in the variegated leaf phenotype [6]. We supported this notion by quantifying the differential phosphorylation levels of PRK in different leaf color sectors. Similarly, the phosphorylation levels of proteins involved in nitrogen metabolism differed significantly between the green and yellow sectors. Compared with green sectors, the phosphorylation levels of glutamine synthases at Ser 175 was higher in the yellow sectors. Glutamine syntheses is a central component of nitrogen metabolism and the phosphorylation of this enzyme is related to nitrogen remobilization [36]. Therefore, we inferred that the phosphorylation of these enzymes was a prerequisite for regulating energy metabolism in different leaf color sectors.

Protein post-modification generally requires upstream enzymes to recognize substrate-specific motifs. Therefore, the recognition and study on the conserved motifs of the modified proteins are important to the prediction of post-translational modifications [37]. We found that the kinase PRK phosphorylated around the motif ...R..S......, phosphoenolpyruvate carboxykinase (PEPCK) could regulate the motif ....P.TP....., 6-phosphofructo-2-kinase/fructose-2,6-bisphosphate 2-phosphatase (PFKFB3) targeted the motif ....S.S...... and the motif ......SP..... could be recognized by putative SNF1-related protein kinase family proteins. These kinases are responsive to glucose levels, and their phosphorylation levels might modulate glycometabolism in different leaf sectors of *Maiyuanjinqiu* [38]. The effect of the phosphorylation sites of these kinases on leaf color remains to be further demonstrated.

### 3.4. DPs Involved in Stress Response and Defense

The phosphorylation levels of proteins associated with stress or stimulus were upregulated in the yellow sectors. The phosphorylation level of L-ascorbate peroxidase 3 was consistent with the increased APX enzyme activity in the yellow sectors (Figure 6 and Figure 8B). This suggested that phosphorylation at this site might positively regulate APX enzyme activity. However, the phosphorylation level of glutathione peroxidase (GPX) related to antioxidant stress was lower in the yellow sectors than in the green sectors (Figure 6). We speculated that the phosphorylation of different antioxidant enzymes might exhibit different regulatory patterns for enzyme activity. The phosphorylation levels of these proteins relative to stress or stimulus might be an adaptation mechanism in the yellow sectors [39]. Furthermore, the enriched pathways of glyoxylate and dicarboxylate metabolism and glycine, serine and threonine metabolism were upregulated in the yellow sectors. These two pathways also could participate in the response to biotic or abiotic stress (Figure 9C) [40,41]. Taken together, the results showing differential phosphorylation levels of stress-related proteins between the yellow and green sectors were remarkable. Our data supported the notion that the yellow/white sectors suffered from photooxidative stress or other stresses [1,42].

### 3.5. DPs Involved in Protein Homeostasis

Previous studies have shown that the homeostasis of protein synthesis and degradation is critical for the formation of variegation [43]. In our study, the phosphorylation levels of the translation initiation factors EIF4G-1, EIF4G and EIF4B were not uniformly upregulated or downregulated in the yellow leaves, and these different phosphorylation sites might have different regulatory effects on protein function (Figure 9D). EIF4G acts as a hub protein together with eIF3 and additional initiation factors, recruiting 40S ribosomal subunits to mRNA [44,45]. The chloroplast translation initiation factors (translation initiation factor 3) could regulate the *var2*-mediated leaf variegation [46,47]. The phosphorylation levels of the 40S ribosomal protein subunits were downregulated in the yellow leaves, while those of the 60S ribosome were upregulated (Figure 9D). This result was contrary to the differential phosphorylation observed in sugarcane mosaic virus infection [48].

Protein hydrolysis or proteolysis is essential for protein homeostasis [49]. The Clp protease system and metalloprotease FTSH are the most important stromal proteases in chloroplast because they are responsible for normal protein turnover and chloroplast development [50]. In the yellow sectors, the phosphorylation levels of ATP -dependent protease Clp protease hydrolysis subunit (Ser 287 and 288) and FTSH1 (Thr 539) were upregulated (Figure 9D). The Clp protease system could control the ALA synthesis through posttranslational regulation of GluTR [51]. The higher phosphorylation level of Clp protease subunits might play a direct or indirect role in chlorophyll biosynthesis. The increased phosphorylation levels of autophagy in the yellow sectors might be related to the phosphorylation of Clp protease [52]. In *Arabidopsis thaliana*, the absence of FTSH1 presents a typical variegated leaf phenotype [53]. The phosphorylation of FTSH1 can change its hydrolytic activity and affect the formation of the photosynthetic complex [54,55]. In the yellow leaves, the phosphorylation levels of other proteins involved in protein degradation or hydrolysis were also upregulated, including putative dynamin homolog, β-glucosidase family protein, glycoside hydrolase family and ubiquitin family proteins (Figure 9D). These differences in protein synthesis and degradation might affect protein homeostasis and leaf color.

### 3.6. DPs Involved in Transcriptional Regulation and Transport

Compared with those in the green sectors, the phosphorylation levels of proteins involved in transcriptional regulation, such as SNF2 and MYB, were globally downregulated in the yellow sectors. In contrast, the phosphorylation levels of zinc finger domains were upregulated in the yellow sectors (Figure 9E). A recent study showed that the SNF2 chromatin-remodeling ATPase enzyme BRM could modulate chlorophyll biosynthesis, and that BRM activity could be controlled by phosphorylation /dephosphorylation [56,57]. The phosphorylated MYB transcription factors could optimize the flavonoid metabolism in soybean under salt stress and could regulate anthocyanin biosynthesis by interacting with bHLH and WD40 [58,59]. We also identified upregulated (1.684-fold) phosphorylation at the Ser 135 site of histone H_2_B in the yellow sectors (Figure 9E). Previous research indicated that the phosphorylation of H_2_B at Ser6 contributed to chromosomal stability [60]. These phosphoproteins involved in transcriptional regulation might play an important role in leaf color. Furthermore, we found that the phosphorylation levels of enriched pathways were all upregulated except for single-organism transport, localization, and transmembrane transport in yellow leaves (Figure 6 and Figure 9A). The differential phosphorylation of proteins in these processes might mediate the direction of sugar transport in *Maiyuanjinqiu*, which was of great significance for the variegated phenotype.

## 4. Materials and Methods

### 4.1. Plant Material

*Maiyuanjinqiu* is a variety derived from *C. fargesii* seedlings. The leaves of *Maiyuanjinqiu* display a variegated phenotype. *Maiyuanjinqiu* and *C. fargesii* were both cultivated in the experimental field (Luoyang, China). The leaves were separately sampled according to the marked positions shown in Figure 1B. The samples were wrapped in tin foil paper, immediately frozen in liquid nitrogen, and then stored at −80 °C until use. The frozen samples were used for the protein extraction and quantitative phosphorylomic analysis.

### 4.2. Determination of Photosynthetic Fluorescence Parameters and Pigment Contents

Potted plants were developed in the greenhouse of Chinese Academy of Forestry. A chlorophyll fluorescence imager (CFI) (Technologica, Essex, UK) was used to obtain the imaging photo of Fv/Fm, following the method of Baker and Oxborough [61] (*n* ≥ 10). ETR(I), ETR(II), and NPQ were detected by a Dual-PAM-100 fluorometer (Walz, Effeltrich, Germany) and the photosynthetically active radiation was set as 129 µmol·m^−2^·s^−1^, which was suitable for the determination of different leaf color sectors (*n* = 18). The third leaves from top to bottom were selected for determination. After the determination, samples were taken for the measurement of pigment contents. The levels of chlorophyll and carotenoid were determined according to the method described by Lichtenthaler [62] (*n* ≥ 4). The lutein content was detected with ELISA Kits (Mlbio, Shanghai, China) (*n* ≥ 8).

### 4.3. Protein Extraction, Trypsin Digestion, and TMT Labeling

The protein extraction was performed by the method of Tong, et al. [63] with some modification. The frozen leaves were ground into a fine powder and sonicated on ice 3 times in lysis buffer. After centrifugation, the obtained supernatant was precipitated with precooled TCA, followed by washing 3 times with cold acetone. The acetone was evaporated at 4 °C, and the powder was redissolved in buffer (8 M urea, 100 mM TEAB, pH = 8). Before the next step, the protein concentration was quantified using a 2-D Quant Kit (GE Healthcare, Piscataway, NJ, USA).

Protein reduction, alkylation, and dilution were performed by the method of Xu, et al. [64]. The protein solution was reduced at 37 °C and alkylated at room temperature. We modified the urea concentration to less than 2 M. For subsequent trypsin digestion and TMT labeling, the method refers to the previously described [63].

### 4.4. HPLC Fractionation and Affinity Enrichment

HPLC fractionation was performed by using an acetonitrile gradient; the 80 fractions obtained by high pH reverse-phase HPLC were combined into 14 components. The phosphopeptides were enriched using IMAC combined with Ti^4+^ enrichment. The Ti^4+^-IMAC material was prepared as described in the previous research [20]. The non-specific phosphopeptides were removed and the obtained phosphopeptides were eluted with 10% NH_4_OH with vibration. The collected phosphopeptides were lyophilized for LC-MS/MS analysis.

### 4.5. LC-MS/MS Analysis

The peptides were dissolved with 0.1% formic acid (Fluka, Buchs, Switzerland) in 2% acetonitrile (Thermo Scientific, San Jose, CA, USA), and then loaded directly onto the reversed-phase analysis column. Intact peptides were detected by tandem mass spectrometry (MS/MS) in an Orbitrap FusionTM TribridTM (Thermo Scientific, San Jose, CA, USA). For a full range mass scan, the resolution was set to 60,000, while the ion fragments were detected at a resolution of 15,000. For MS/MS, 35% normalized collision energy (NCE) was applied. The threshold of precursor ions was greater than 5E3, and the dynamic exclusion was set to 15.0 s. One MS scan was alternated sequentially with 20 scans. Accumulated 5E4 ions were used for the whole MS/MS spectra of the phosphorylome. The MS scan range was 350 to 1550 m/z, and the fixed first mass was 100 m/z.

### 4.6. Database Search and Data Analysis

The spectrometric data were searched by MaxQuant software with the Andromeda search engine (v.1.5.2.8, Martinsried, Germany). Tandem mass spectra were matched against the *Catalpa bungei* protein database) [65]. The false discovery rate (FDR) was specified as 1%. The site location probability was greater than 0.5. The significant fold-change cutoff was 1.5-fold for differential proteins in the Y1 versus G1, Y1 versus Y2 and Y2 versus G2 comparisons. The fold changes of phosphorylated proteins were normalized to the protein fold change in proteome [66]. The raw phosphoproteomics data was deposited in the ProteomeXchange with identifier PXD013065.

### 4.7. Bioinformatics Methods

Soft motif-x was used to analyze the upstream and downstream amino acids of the modified site in all the phosphoproteins. Functional enrichments were tested using a two tailed Fisher’s exact test, and a corrected *p* value < 0.05 was considered significant. Subcellular localization prediction was conducted by the updated version of WOLFPSORT (http://www.genscript.com/wolf-psort.html). GO annotation of the phosphoproteins was derived from the UniProt-GOA database (http://www.ebi.ac.uk/GOA/). InterProScan was used to annotate the unmapped proteins using the protein sequence. The identified protein domain descriptions were annotated by the InterPro database (http://www.ebi.ac.uk/interpro/). To better understand the mechanism of why the leaves had two colors with the same genetic background, we focused on the DPs in the Y1 versus Y2 comparison for KEGG pathway analysis, removing the DPs in the G1 versus G2 comparison. To understand which organelles play a key role in the formation of variegation, the analysis was based on the chloroplast, cytosol, nuclear and mitochondria. The KEGG database (http:// www.genome.jp/kegg/) was used to annotate the protein pathways. PPIs were predicted by STRING database version 10.5 (http://string-db.org/). The filtering scores was set as ≥0.7 (high confidence), and the mapping organism was *Populus trichocarpa*. The PPIs of the DPs in different comparisons were visualized by the Cytoscape software.

### 4.8. Key Enzyme Activities

The enzyme activities of ALAD, PBGD, CPOX, PPOX, SOD, and APX were measured following the protocol described in the instructions of ELISA Kits (Mlbio, Shanghai, China).

## 5. Conclusions

This study is the first to dissect the differences in global phosphorylation levels in different leaf color sectors in woody plants. As a result, 1434 phosphoproteins were quantified, among which, 165 phosphoproteins were specifically related to the color sectors in *Maiyuanjinqiu*. Integrative bioinformatics analysis revealed that pigment biosynthesis, photosynthesis, and energy metabolism, protein homeostasis, stress response, transcriptional regulation, and transport were the key pathways contributing to variegation. KEGG pathway enrichment suggested that the enzymes located in the chloroplasts and cytosol might play a central role in the variegated phenotype. The phosphorylation function of kinases, such as PRK, PEPCK, PFKFB3, and SNF1-related protein kinase family proteins, needs to be further researched. Similarly, further functional studies of the role of FTSH1 phosphorylation in leaf development would be of great significance. In addition, physiological and biochemical measurements confirmed that protein phosphorylation could affect enzyme activities and plant physiology.

## Supplementary Materials

Supplementary materials can be found at https://www.mdpi.com/1422-0067/20/8/1895/s1.

## Abbreviations

Fv/Fm	Maximum quantum yield of PSII
ETR	Electron transport rate
NPQ	Non photochemical quenching
PS	Photosystem
LC-MS/MS	Liquid chromatography–mass spectrometry/mass spectrometry
GO	Gene ontology
DPs	Differential phosphoproteins
LHCB	Chlorophyll a-b binding protein
ATPF 1G	ATP synthase subunit gamma
PsbO	Oxygen-evolving enhancer protein 1
PsbP	Oxygen-evolving enhancer protein 2
PsbQ	Oxygen-evolving enhancer protein 3
PsbR	Photosystem II reaction center protein H
PsbH	Photosystem II reaction center protein H
LHCB	Chlorophyll a-b binding protein
PsaE	Putative Photosystem I reaction center subunit IV
PsaF	Putative photosystem I reaction center subunit III
PC	Plastocyanin
GluTR	Glutamyl-tRNA reductase
NCED	9-cis-epoxycarotenoid dioxygenase 1
P450	Cytochrome P450 family protein, expressed
3.1.3.37	Putative fructose-1,6-bisphosphatase
PRK	Phosphoribulokinase
rbcL	Ribulose bisphosphate carboxylase large chain
ALDO	Fructose-bisphosphate aldolase
GAPA	EST C74302 (E30840) corresponds to a region of the predicted gene
GAPDH	Glyceraldehyde-3-phosphate dehydrogenase 2, cytosolic
PGK	Phosphoenolpyruvate carboxykinase
PK	Pyruvate kinase
gpml	Putative 2, 3-bisphosphoglycerate-independent phosphoglycerate mutase
glgC	Glucose-1-phosphate adenylyltransferase
Glu	Glutamine syntheses
TrpA	Tryptophan synthase alpha chain
GLDC	Putative glycine dehydrogenase
GGAT	Alanine aminotransferase
gcvT	Aminomethyltransferase
AGXT	Serine-glyoxylate aminotransferase
asd	Aspartate-semialdehyde dehydrogenase family protein, expressed
SNF2	SNF2 transcription factor
MYB	MYB transcription factor
H2B	Histone H2B
APX	Ascorbate peroxidase
RP	Ribosomal protein
EIF	Eukaryotic translation initiation factor
Clp	ATP-dependent Clp protease proteolytic subunit
FTSH1	ATP-dependent zinc metalloprotease FTSH 1, chloroplastic
DNM	Putative dynamin homolog
GIDIL	Gibberellin receptor GID1L2
lacZ	Glycoside hydrolase family 2 family protein
PMA	H-ATPase
ALAD	ALA dehydrogenase
PBGD	Porphobilinogen deaminase
CPOX	Coproporphyrinogen III oxidase
PPOX	Protoporphyrinogen IX oxidase
SOD	Superoxide dismutase
ELISA	Enzyme-linked immunosorbent assay

## Appendix A

**Table A1:** The phosphorylation modification levels of the key proteins on the enriched pathways based on functional enrichment analysis in G1, G2, Y1 and Y2.

Protein Description (Abbreviation/Phosphorylated Amino Acid/Site)	G1	G2	Y1	Y2
**Pigment biosynthesis**				
Glutamyl-tRNA reductase (GluTR/S/55)	0.53	0.69	2.37	0.92
Porphobilinogen deaminase, chloroplastic (hemC/T/339)	0.72	0.78	1.34	1.17
9-cis-epoxycarotenoid dioxygenase 1 (NCED/S/51)	0.42	0.44	1.66	1.84
Cytochrome P450 family protein, expressed (P450/S/251)	0.53	0.74	1.17	1.52
Cytochrome P450 family protein, expressed (P450/S/134)	0.46	0.69	1.08	1.62
**Photosynthesis**				
Oxygen-evolving enhancer protein 1 (PsbO/Y/252)	0.26	0.34	3.29	0.95
Oxygen-evolving enhancer protein 1 (PsbO/S/239)	0.35	0.48	3.12	0.84
Oxygen-evolving enhancer protein 1 (PsbO/S/174)	0.20	0.27	3.02	0.88
Oxygen-evolving enhancer protein 1 (PsbO/S/173)	0.40	0.46	2.73	0.76
Oxygen-evolving enhancer protein 1 (PsbO/S/141)	0.50	0.67	2.65	0.80
Oxygen-evolving enhancer protein 1 (PsbO/Y/187)	0.37	0.48	2.64	0.83
Oxygen-evolving enhancer protein 1 (PsbO/S/88)	0.77	0.99	1.46	0.90
Oxygen-evolving enhancer protein 1 (PsbO/S/153)	0.77	0.82	1.35	1.18
Oxygen-evolving enhancer protein 1 (PsbO/S/249)	0.86	0.97	1.34	0.95
Oxygen-evolving enhancer protein 1 (PsbO/S/238)	0.16	0.21	3.65	0.96
Oxygen-evolving enhancer protein 2 (PsbP/S/50)	0.26	0.30	5.13	0.94
Oxygen-evolving enhancer 2-1, chloroplastic (PsbP/Y/21)	0.89	0.95	1.71	1.04
Oxygen-evolving enhancer 2-1, chloroplastic (PsbP/T/22)	0.89	0.94	1.64	1.07
Oxygen-evolving enhancer protein 3-1 (PsbQ/T/123)	0.84	0.81	2.54	1.28
Oxygen-evolving enhancer protein 3-1 (PsbQ/Y/118)	0.82	0.92	1.30	1.39
Photosystem II reaction center protein H (PsbH/T/3)	1.05	1.18	0.85	0.76
Photosystem II reaction center protein H (PsbH/T/5)	0.95	1.19	2.08	0.68
Photosystem II 10 kDa polypeptide (PsbR/Y/74)	0.79	1.13	1.64	0.93
Plastocyanin (PetE/S/154)	0.79	0.88	1.32	0.85
Putative photosystem I reaction center subunit III (PsaF/S/100)	0.69	0.78	3.53	1.38
Putative Photosystem I reaction center subunit IV (PsaE/S/93)	0.42	0.50	3.46	1.37
Putative Photosystem I reaction center subunit IV (PsaE/S/101)	0.74	0.77	2.31	1.05
Putative photosystem I reaction center subunit III (PsaF/S/99)	0.69	0.78	3.39	1.35
ATP synthase subunit gamma (ATPF1G/S/348)	0.53	0.65	3.33	1.13
ATP synthase subunit gamma (ATPF1G/Y/351)	0.55	0.66	3.28	1.11
ATP synthase subunit gamma (ATPF1G/S/99)	0.55	0.66	3.28	1.11
**Photosynthesis –antenna proteins**				
Chlorophyll a/b-binding protein (LHCB4/S/49)	0.94	0.97	2.15	0.91
Chlorophyll a/b-binding protein (LHCB4/S/49)	0.94	0.97	2.15	0.91
Chlorophyll a-b binding protein 2, chloroplastic (LHCB1/S/38)	0.76	0.89	3.90	0.90
Chlorophyll a-b binding protein 1(LHCB1/T/23)	0.76	0.89	1.73	1.01
Chlorophyll a-b binding protein 1 (LHCB1/S/43)	0.48	0.59	1.66	1.97
Chlorophyll a-b binding protein 1 (LHCB1/Y/49)	1.14	1.09	0.59	0.84
Chlorophyll a-b binding protein 2, chloroplastic (LHCB1/S/47)	1.01	1.00	0.45	1.01
Chlorophyll a-b binding protein 1 (LHCB1/S/41)	1.11	1.08	0.42	1.00
Chlorophyll a-b binding protein 2, chloroplastic (LHCB1/Y/50)	1.05	0.97	0.27	1.01
**Stress and stimulus response proteins**				
Catalase (katE/S/347)	0.52	0.59	2.23	1.12
Catalase (katE/S/349)	0.49	0.70	2.16	1.03
Catalase (katE/S/112)	0.55	0.66	2.00	1.15
Catalase (katE/T/115)	0.59	0.65	1.95	1.14
Catalase (katE/S/11)	0.78	0.85	1.35	0.98
Catalase (katE/T/351)	0.47	0.59	1.85	1.41
Probable L-ascorbate peroxidase 3 (E1.11.1.11/S/150)	0.70	0.74	2.19	1.25
OSJNBb0048E02.12 protein (OSJNBb0048E02.12/S/8)	0.62	0.78	1.44	0.89
Glutaredoxin domain containing protein (S/154)	0.66	1.02	1.43	0.94
OSJNBb0048E02.12 protein (OSJNBb0048E02.12/S/50)	0.80	0.77	1.40	0.81
OSJNBb0048E02.12 protein (OSJNBb0048E02.12/Y/3)	0.66	0.78	1.39	0.96
OSJNBb0048E02.12 protein (OSJNBb0048E02.12/S/5)	0.68	0.8	1.36	0.98
Putative cinnamyl-alcohol dehydrogenase family protein (E1.1.1.195/T/7)	1.00	1.08	1.39	0.90
Glutaredoxin domain containing protein (S/99)	0.87	1.00	1.38	0.79
Glutaredoxin domain containing protein (S/46)	0.87	1.06	0.79	1.27
Probable cinnamyl alcohol dehydrogenase 3 (S/20)	0.90	1.03	1.38	0.83
Glutaredoxin domain containing protein (S/103)	0.86	1.00	1.34	0.84
Putative nuclear snf4-like (S/48)	0.91	0.98	1.32	0.87
Homeobox-leucine zipper protein ROC1 (S/46)	1.01	1.08	4.2	0.40
Lipoxygenase (LOX2S/S/76)	0.46	0.59	2.71	1.31
Lipoxygenase (LOX2S/S/74)	0.44	0.51	2.7	1.5
Putative ENTH/ANTH/VHS superfamily protein isoform 1 (S/180)	0.78	0.81	1.78	0.71
Integral membrane single C2 domain protein (T/475)	0.83	0.90	1.66	0.73
Integral membrane single C2 domain protein (T/473)	0.82	0.93	1.64	0.73
Clathrin assembly protein AP180 short form-like (S/306)	0.84	0.91	1.62	0.78
Clathrin assembly protein AP180 short form-like (T/307)	0.93	0.94	1.42	0.77
Putative ENTH/ANTH/VHS superfamily protein isoform 1 (T/221)	0.91	0.93	1.26	0.78
Peroxisomal (S)-2-hydroxy-acid oxidase GLO5 (HAO/T/159)	0.97	1.14	1.22	0.79
Monodehydroascorbate reductase (E1.6.5.4/S/344)	0.74	1.33	1.17	0.74
Monodehydroascorbate reductase (E1.6.5.4/T/346)	0.69	1.30	1.16	0.86
Chaperonin (Chaperonin/T/125)	0.73	0.81	1.09	1.25
Glutathione peroxidase (gpx/S/129)	0.98	1.19	0.75	1.33
Putative C2 domain-containing protein (S/406)	0.87	1.05	0.71	1.32
L-lactate dehydrogenase (LDH/S/26)	1.22	1.24	0.75	0.81
**Energy and metabolism**				
Fructose-bisphosphate aldolase (ALDO/T/88)	0.67	0.67	3.48	0.97
Fructose-bisphosphate aldolase (ALDO/S/42)	0.56	0.70	2.72	0.78
Fructose-bisphosphate aldolase (ALDO/S/82)	0.67	0.88	1.68	0.91
Fructose-bisphosphate aldolase (ALDO/S/73)	0.76	1.06	1.44	0.93
Fructose-bisphosphate aldolase (ALDO/S/83)	0.79	0.91	1.44	0.82
Fructose-bisphosphate aldolase (ALDO/S/349)	0.92	0.99	1.42	0.76
Fructose-bisphosphate aldolase (ALDO/T/80)	0.83	0.96	1.36	1.13
Transketolase isoform 1 (transketolase [EC:2.2.1.1]/Y/426)	0.66	0.67	3.35	0.77
Transketolase isoform 1(transketolase ([EC:2.2.1.1]/S/434)	0.77	0.89	1.76	0.78
Transketolase isoform 1 ( transketolase [EC:2.2.1.1]/S/430)	0.97	1.14	1.22	0.83
EST C74302(E30840) corresponds to a region of the predicted gene (GAPA/S/26)	0.40	0.57	2.35	0.79
EST C74302(E30840) corresponds to a region of the predicted gene (GAPA/S/27)	0.48	0.59	2.27	0.82
EST C74302(E30840) corresponds to a region of the predicted gene (GAPA/S/25)	0.75	0.82	1.52	0.81
EST C74302(E30840) corresponds to a region of the predicted gene (GAPA/S/278)	0.81	0.90	1.37	1.14
EST C74302(E30840) corresponds to a region of the predicted gene (GAPA/T/295)	0.90	1.29	1.12	0.99
putative 2,3-bisphosphoglycerate-independent phosphoglycerate mutase (gpmI/S/81)	0.84	0.88	1.53	0.77
Phosphoenolpyruvate carboxykinase (ATP), conserved site-containing protein (E4.1.1.49/T/71)	0.76	1.06	1.44	0.66
Phosphoglucomutase/phosphomannomutase, C-terminal domain, putative (pgn/T/5)	0.81	0.86	1.27	0.75
Carbonic anhydrase (cynT/S/111)	0.51	0.66	2.14	0.75
Carbonic anhydrase (cynT/S/109)	0.80	0.86	1.19	0.78
Carbonic anhydrase (cynT/S/83)	0.84	1.01	1.52	1.26
Phosphoribulokinase (PRK/T/78)	0.60	0.60	2.03	0.85
Phosphoribulokinase (PRK/S/219)	0.64	0.80	1.45	0.94
Phosphoribulokinase (PRK/S/79)	0.88	1.03	1.45	0.85
Putative fructose-1,6-bisphosphatase (E3.1.3.37/S/320)	0.72	0.82	1.80	0.83
Serine-glyoxylate aminotransferase (AGXT/Y/35)	0.85	0.87	1.76	1.02
Serine-glyoxylate aminotransferase (AGXT/S/37)	0.69	0.73	1.46	1.00
Ribulose bisphosphate carboxylase small chain (rbcS/S/114)	0.7	1.24	1.59	1.16
Ribulose bisphosphate carboxylase small chain (rbcS/Y/118)	1.01	1.00	1.37	1.21
Ribulose bisphosphate carboxylase small chain (rbcS/Y/117)	0.95	1.26	1.21	1.20
GLO5 Peroxisomal (S)-2-hydroxy-acid oxidase GLO5 (HAO/T/159)	0.79	0.97	1.42	0.79
2-oxoglutarate dehydrogenase, putative (OGDH/S/344)	0.94	1.01	1.33	0.91
Putative fructose-1,6-bisphosphatase (E3.1.3.37/S/318)	0.88	0.89	1.31	1.10
Phosphoglycerate kinase (PGK/S/276)	0.83	0.94	1.26	1.05
Aminomethyltransferase (gcvT/S/268)	0.75	0.94	2.09	0.92
Aminomethyltransferase (gcvT/Y/390)	1.02	1.22	1.10	1.10
Aminomethyltransferase (gcvT/S/339)	0.74	1.02	0.83	0.82
Glucose-1-phosphate adenylyltransferase (glgC/S/263)	0.39	0.41	2.13	1.10
Glucose-1-phosphate adenylyltransferase (glgC/S/424)	0.48	0.47	2.05	1.05
UDP-glucose 6-dehydrogenase (UGDH/S/393)	1.15	1.12	0.69	0.96
1,3-beta-glucan synthase component-like (S/1019)	0.80	1.04	1.43	0.79
Putative beta 1,3 glucan synthase (T/27)	0.76	1.05	1.41	0.91
Serine hydroxymethyltransferase (glyA/S/384)	0.72	0.82	1.8	1.06
Phospho-2-dehydro-3-deoxyheptonate aldolase 1, chloroplastic (E2.5.1.54/S/67)	0.43	0.47	1.72	1.10
Aspartate-semialdehyde dehydrogenase family protein, expressed (ASD/S/137)	0.72	0.79	1.65	0.78
Orange pericarp1 (trpB/S/76)	0.79	0.97	1.48	0.91
Cellulose synthase-like protein E6 (IPR029044/S/393)	0.5	0.63	1.8	1.36
Glyceraldehyde-3-phosphate dehydrogenase 2, cytosolic (GAPDH/Y/257)	0.75	0.82	1.52	0.74
Putative beta 1,3 glucan synthase (S/29)	0.81	1.08	1.29	0.90
putative 6-phosphofructo-2-kinase / fructose-2, 6-bisphosphate 2-phosphatase(S/276)	1.04	1.16	0.56	1.28
Glutamine synthetase (Gs/S/175)	0.76	0.87	1.61	0.98
Putative glycine dehydrogenase (GLDC/S/51)	0.77	0.89	1.76	0.95
Thiamine thiazole synthase, chloroplastic (THI4/S/27)	0.37	0.37	2.41	0.62
Nitrogen regulatory protein P-II homolog (/T114)	0.51	0.62	1.71	0.63
Nitrogen regulatory protein P-II homolog (/T113)	0.57	0.7	1.61	0.68
Nitrate-induced NOI protein, expressed (RIN4/T/161)	0.61	0.96	1.53	0.70
Nitrate-induced NOI protein, expressed (RIN4/T/206)	0.77	1.13	1.34	0.64
Nitrate-induced NOI protein, expressed (RIN4/T/216)	0.74	1.16	1.32	0.68
Nitrate-induced NOI protein, expressed (RIN4/T/227)	0.79	1.07	1.22	0.88
Nitrate-induced NOI protein, expressed (RIN4/T/168)	0.77	0.78	1.54	0.63
Nitrate-induced NOI protein, expressed (RIN4/S/170)	0.82	0.74	1.39	0.68
Nitrate-induced NOI protein, expressed (RIN4/S/96)	0.96	0.95	1.31	0.66
Nitrate-induced NOI protein, expressed (RIN4/T/165)	0.79	0.88	1.4	0.75
Nitrate-induced NOI protein, expressed (RIN4/T/228)	0.86	0.87	1.32	0.82
Nitrate-induced NOI protein, expressed (RIN4/S/154)	0.99	1.03	1.19	0.77
Nitrate-induced NOI protein, expressed (RIN4/S/183)	0.87	0.91	1.28	0.84
Nitrate-induced NOI protein, expressed (RIN4/S/127)	0.93	0.96	1.23	0.81
Nitrate-induced NOI protein, expressed (RIN4/S/164)	0.83	0.92	1.28	0.85
Nitrate-induced NOI protein, expressed (RIN4/S/225)	0.72	1.31	1.09	0.92
Nitrate-induced NOI protein, expressed (RIN4/S/133)	1.22	1.16	0.97	0.77
Nitrate-induced NOI protein, expressed (RIN4/S/185)	1.59	1.84	0.4	0.43
Nitrate-induced NOI protein, expressed (RIN4/S/95)	1.17	1.39	0.75	0.84
**Protein synthesis**				
50S ribosomal protein L21 (RP-L21/S/52)	0.32	0.38	2.4	1.18
60S acidic ribosomal protein P0 (RP-LP0/S/311)	0.25	0.35	1.53	0.25
60S acidic ribosomal protein P1 (RP-LP1/S/103)	0.23	0.21	1.51	0.28
60S ribosomal protein L6 (RP-L6e/S/204)	0.9	0.92	1.36	0.86
60S acidic ribosomal protein P2A (RP-LP2/S/105)	0.15	0.34	1.33	0.15
60S acidic ribosomal protein P3 (RP-LP1/S/110)	0.35	0.47	1.31	0.37
60S ribosomal protein L24 (RP-L24e/S/28)	0.82	1.09	1.24	0.86
40S ribosomal protein S27 (RP-S27e/S/80)	1.15	1.70	0.65	0.80
40S ribosomal protein S6 (RP-S6e/S/231)	1.00	1.17	0.65	0.96
40S ribosomal protein S6 (RP-S6e/S/237)	1.02	1.3	0.51	0.76
40S ribosomal protein S6 (RP-S6e/S/229)	1.21	1.32	0.29	0.56
Putative translational activator (S/1831)	0.78	0.83	1.88	0.78
4G-1 Eukaryotic translation initiation factor isoform 4G-1 (EIF4G/S/527)	0.86	1.11	1.34	0.74
4B Eukaryotic translation initiation factor 4B (EIF4B/S/128)	0.72	0.84	1.29	0.86
3H Eukaryotic translation initiation factor 3 subunit H (EIF3H/S/222)	0.96	1.27	0.91	0.79
4G Eukaryotic translation initiation factor 4G (EIF4G/T/1579)	1.49	1.52	0.8	1.15
4B Eukaryotic translation initiation factor 4B (EIF4B/Y/536)	1.15	1.12	0.73	1.17
4G Eukaryotic translation initiation factor 4G (EIF4G/S/184)	1.74	1.53	0.73	1.13
4G Eukaryotic translation initiation factor 4G (EIF4G/S1401)	2.24	1.76	0.59	1.00
Elongation factor 1-alpha (EEF1A/S/176)	1.22	1.07	0.54	1.18
**Protein Degradation**	G1	G2	Y1	Y2
Putative dynamin homolog (DNM/S/894)	0.47	0.57	2.53	1.44
Putative dynamin homolog (DNM/S/897)	0.52	0.60	2.49	1.38
Putative dynamin homolog (DNM/S/887)	0.77	0.84	1.67	0.80
Putative dynamin homolog (DNM/Y/886)	0.69	0.88	1.67	0.75
Putative dynamin homolog (DNM/T/736)	0.79	0.88	1.56	1.11
Putative dynamin homolog (DNM/S/733)	0.80	0.86	1.49	1.20
Putative dynamin homolog (DNM/S/730)	0.80	0.87	1.48	1.19
Putative dynamin homolog (DNM/T/535)	0.88	0.95	1.32	1.00
Dynamin-related protein 1C, putative, expressed (DNM/S/271)	0.76	0.82	1.66	1.06
ATP-dependent Clp protease proteolytic subunit (ClpP/S/288)	0.31	0.37	2.46	0.89
ATP-dependent Clp protease proteolytic subunit (ClpP/S/287)	0.30	0.46	2.23	0.90
ATP-dependent zinc metalloprotease FTSH 1, chloroplastic (FTSH1/T/539)	0.39	0.53	2.42	0.90
ATP-dependent Clp protease proteolytic subunit (ClpP/S/287)	0.30	0.46	2.23	0.90
ATP-dependent Clp protease proteolytic subunit (ClpP/S/96)	0.49	0.60	1.70	1.00
H-ATPase (PMA1/Y/955)	0.83	0.40	2.02	1.07
H-ATPase (PMA1/T/956)	0.67	0.80	2.01	0.79
Pleiotropic drug resistance protein 5 (S/26)	0.56	0.77	1.83	0.98
Pleiotropic drug resistance protein 6 (S/14)	0.94	1.05	1.28	0.73
Pleiotropic drug resistance protein 6 (S/12)	0.93	1.08	1.21	0.79
Chloroplast thylakoidal processing peptidase-like protein (S/392)	0.54	0.62	1.65	1.11
Neutral/alkaline invertase (Neutral/alkaline invertase/S/48)	0.81	0.99	1.62	0.85
Beta-glucosidase 11 (Beta-glucosidase 11/S546)	0.57	0.60	1.62	0.99
Endonuclease III homologue (NTH/S/346)	0.80	0.82	1.61	0.83
Gibberellin receptor GID1L2 (GID1L2/S/309)	0.62	0.71	1.60	1.25
Beta-glucosidase 11 (Beta-glucosidase 11/S545)	0.60	0.62	1.58	0.98
Beta-glucosidase 11 (Beta-glucosidase 11/S555)	0.67	0.72	1.50	0.93
Beta-glucosidase 11 (Beta-glucosidase 11/S/1007)	0.69	0.77	1.45	1.13
Beta-glucosidase 11 (Beta-glucosidase 11/S/249)	0.70	0.78	1.25	1.21
Beta-glucosidase 24 (Beta-glucosidase 24/S/279)	0.66	0.64	1.47	0.8
Beta-glucosidase 24 (Beta-glucosidase 24 /Y/286)	0.64	0.63	1.47	0.81
Beta-glucosidase 24 (Beta-glucosidase 24/S/271)	0.66	0.83	1.35	0.87
Beta-glucosidase 24 (Beta-glucosidase 24/S/269)	0.66	0.73	1.33	0.94
DNA topoisomerase I (DNA topoisomerase I/S/139)	0.90	0.98	1.41	0.89
DNA topoisomerase I (DNA topoisomerase I/S/142)	0.90	0.98	1.41	0.89
Glycoside hydrolase family 2 family protein (lacZ/S/567)	0.68	0.74	1.35	1.35
Ubiquitin family protein (Ubiquitin family protein/T/351)	0.93	0.92	1.33	0.74
H2B Filamentation temperature-sensitive H2B (S/198)	0.81	1.02	1.24	0.95
H2B Filamentation temperature-sensitive H2B (S/199)	0.81	1.02	1.24	0.95
putative endo-1,3;1,4-beta-D-glucanase (S/209)	0.93	1.06	1.23	0.81
Beta-glucosidase-like SFR2, chloroplastic (S/132)	0.75	1.11	0.85	1.30
Soluble inorganic pyrophosphatase (ppa/S/33)	0.82	1.17	0.77	1.25
Soluble inorganic pyrophosphatase (ppa/S/34)	0.80	1.10	0.77	1.33
DNA topoisomerase I (DNA topoisomerase I/S/192)	1.11	1.21	0.71	0.63
CER5 (CER5/S/330)	1.01	1.24	0.64	1.09
**Transcriptional regulation**				
Histone H2B (H2B/S/135)	0.92	1.02	1.30	0.77
Histone H2B (H2B/S/19)	1.25	1.12	0.73	0.9
SNF2 transcription factor (MSI/S/95)	1.28	1.25	0.72	1.16
MYB transcription factor (S/184)	1.15	1.19	0.66	1.06
Putative MYB DNA-binding domain superfamily protein (S/171)	0.75	1.02	1.25	1.01
Zinc finger CCCH domain-containing protein 8 (S/436)	1.36	1.2	0.82	0.78
Zinc finger CCCH domain-containing protein 8 (S/238)	1.27	1.34	0.59	1.01
Zinc-finger homeodomain protein 1 (S/199)	0.70	0.86	1.30	1.24
Zinc-finger homeodomain protein 1 (T/37)	0.78	1.04	1.22	1.00
Zinc-finger homeodomain protein 1 (T/17)	1.01	0.88	1.40	0.73
Zinc-finger homeodomain protein 3 (Y/151)	0.76	1.00	1.33	1.03
Zinc finger CCCH domain-containing protein 8 (S/436)	1.36	1.20	0.82	0.78
Zinc finger CCCH domain-containing protein 8 (S/238)	1.27	1.34	0.59	1.01
Zinc finger CCCH domain-containing protein 63 (S/408)	0.69	0.97	1.84	0.85
Zinc finger CCCH domain-containing protein 63 (S/410)	0.72	1.01	1.80	0.81
Zinc finger CCCH domain-containing protein 63 (S/183)	0.76	1.04	1.76	0.73
Zinc finger CCCH domain-containing protein 63 (T/185)	0.89	1.08	1.48	0.72
Zinc finger CCCH domain-containing protein 63 (S/424)	0.90	0.93	1.38	0.93
ROC1 Homeobox-leucine zipper protein ROC1 (ROC1/S/46)	1.01	1.08	4.20	0.40
DNA binding protein, mRNA (S/176)	0.94	0.92	0.75	1.28
DNA binding protein, mRNA (S/204)	0.8	0.92	1.33	1.07
DNA binding protein, mRNA (S/64)	1.11	1.09	0.69	1.02
**Transport**				
Hexose transporter (Hexose transporter/S/643)	0.55	0.64	2.11	0.86
Hexose transporter (Hexose transporter/S/70)	0.61	0.75	1.78	0.97
Putative sulfate transporter (S/643)	0.68	0.96	1.90	0.68
Putative sulfate transporter (Putative sulfate transporter/S/651)	0.73	0.9	1.69	0.82
Sugar transport1 isoform 1 (Sugar transport1 isoform 1/S/33)	0.66	0.77	1.56	0.66
Sugar transport1 isoform 1 (Sugar transport1 isoform 1/S/32)	0.84	0.98	1.31	0.75
Sugar transport1 isoform 1 (Sugar transport1 isoform 1/S/485)	1.24	1.32	0.65	1.16
Amino acid permease-like (Amino acid permease-like/S/20)	0.22	0.47	0.98	2.45
Amino acid permease-like (Amino acid permease-like/S/47)	1.10	1.25	0.61	1.03
Amino acid permease-like (Amino acid permease-like/Y/49)	1.25	1.22	0.52	1.02
Amino acid permease-like (Amino acid permease-like /T/41)	1.26	1.23	0.47	1.01
Amino acid permease-like (Amino acid permease-like/S/42)	1.28	1.22	0.45	1.01
Calcium-transporting ATPase 1, plasma membrane-type (S/45)	0.87	1.17	0.73	1.15
MATE efflux family protein (MATE efflux family protein/S/12)	1.10	1.07	0.65	1.01
ABC transporter family protein, putative, expressed (S/99)	0.69	0.89	2.43	0.61
ABC transporter family protein, putative, expressed (S/7)	0.70	0.92	2.36	0.61
ABC transporter family protein, putative, expressed (S/63)	0.81	0.72	2.30	0.72
ABC-2 type transporter family protein (S/617)	0.75	0.80	1.53	1.13
