# Supplementary material for: Quantitative Phosphoproteomic and Physiological Analyses Provide Insights into the Formation of the Variegated Leaf in Catalpa fargesii
Source: Int J Mol Sci. 2019 Apr 17;20(8):1895. doi: 10.3390/ijms20081895 (PMC6514904; doi:10.3390/ijms20081895)
Supplement: Supplementary file 1 [file ijms-20-01895-s001.pdf]

Supplementary Materials:

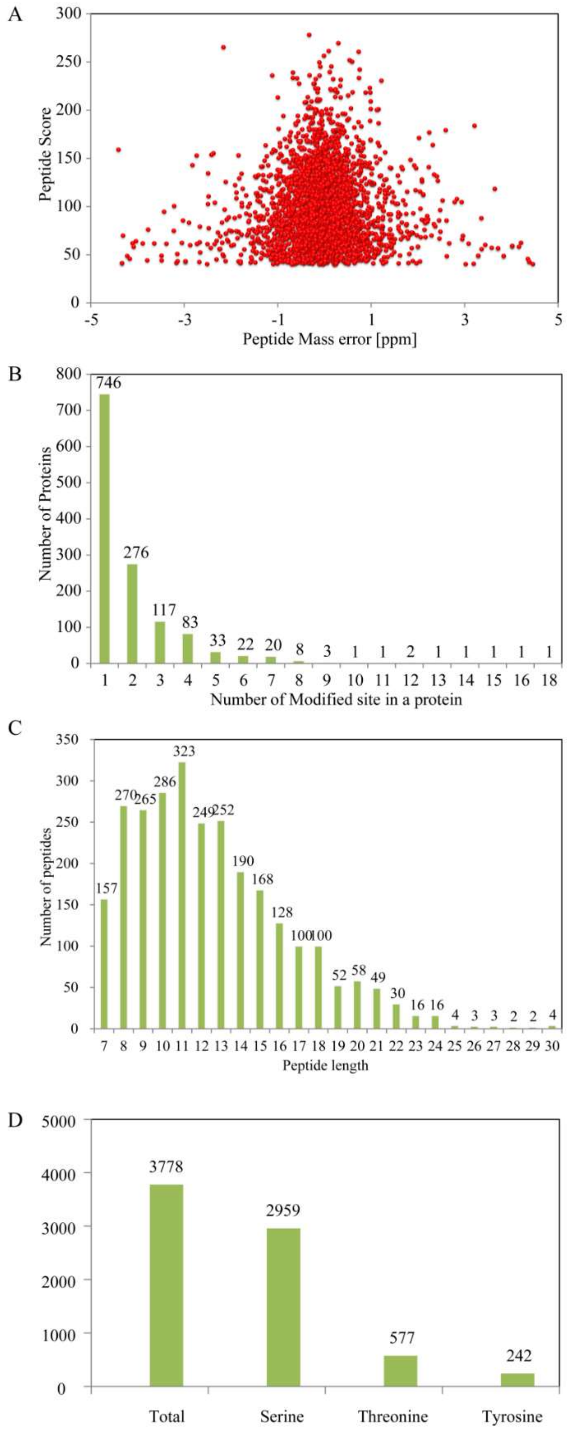

Figure S1. Distribution of phosphorylated peptides and phosphorylated sites identified. (A) Volcano map of error rate distribution by mass spectrometry; (B) length distribution of phosphorylated peptides; (C) distribution of phosphorylation sites; (D) distribution of phosphorylation sites at serine, Threonine and tyrosine residues. Three biological replicates were performed.

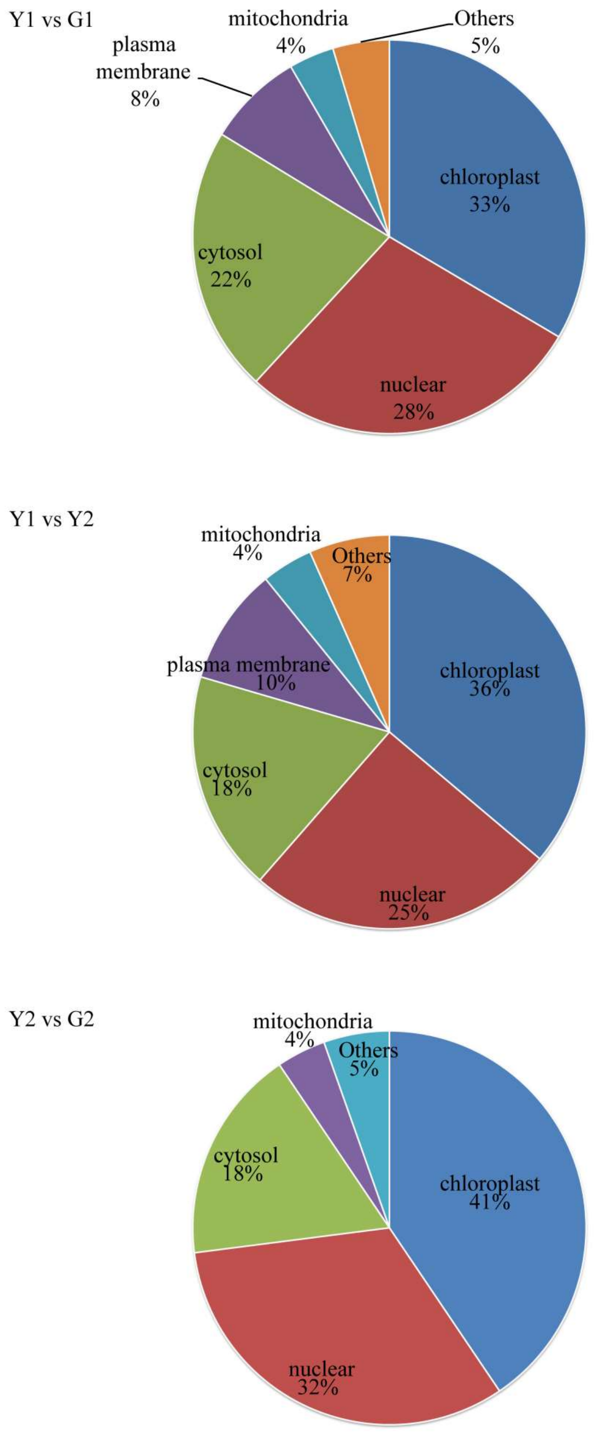

Figure S2. Biological process of GO function classifications on the phosphorylated proteins in the Y1 vs G1, Y1 vs Y2 and Y2 vs G2 comparisons, respectively.

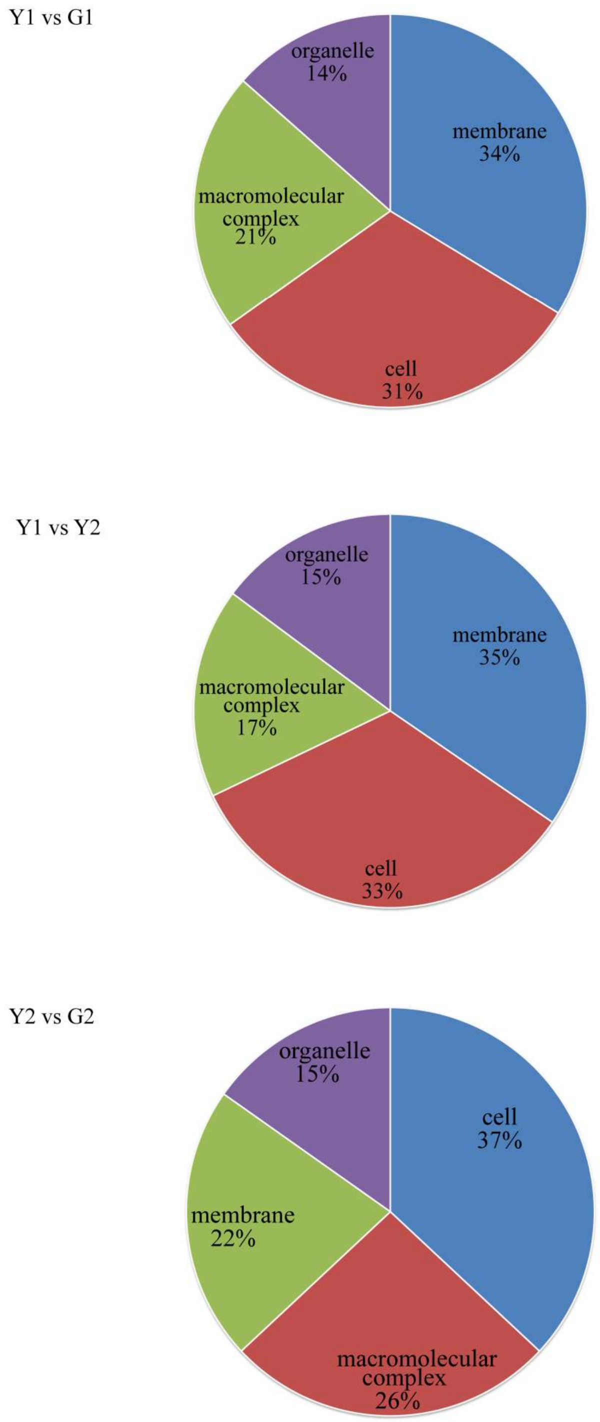

Figure S3. Cellular component of GO function classifications on the phosphorylated proteins in the Y1 vs G1, Y1 vs Y2 and Y2 vs G2 comparisons, respectively.

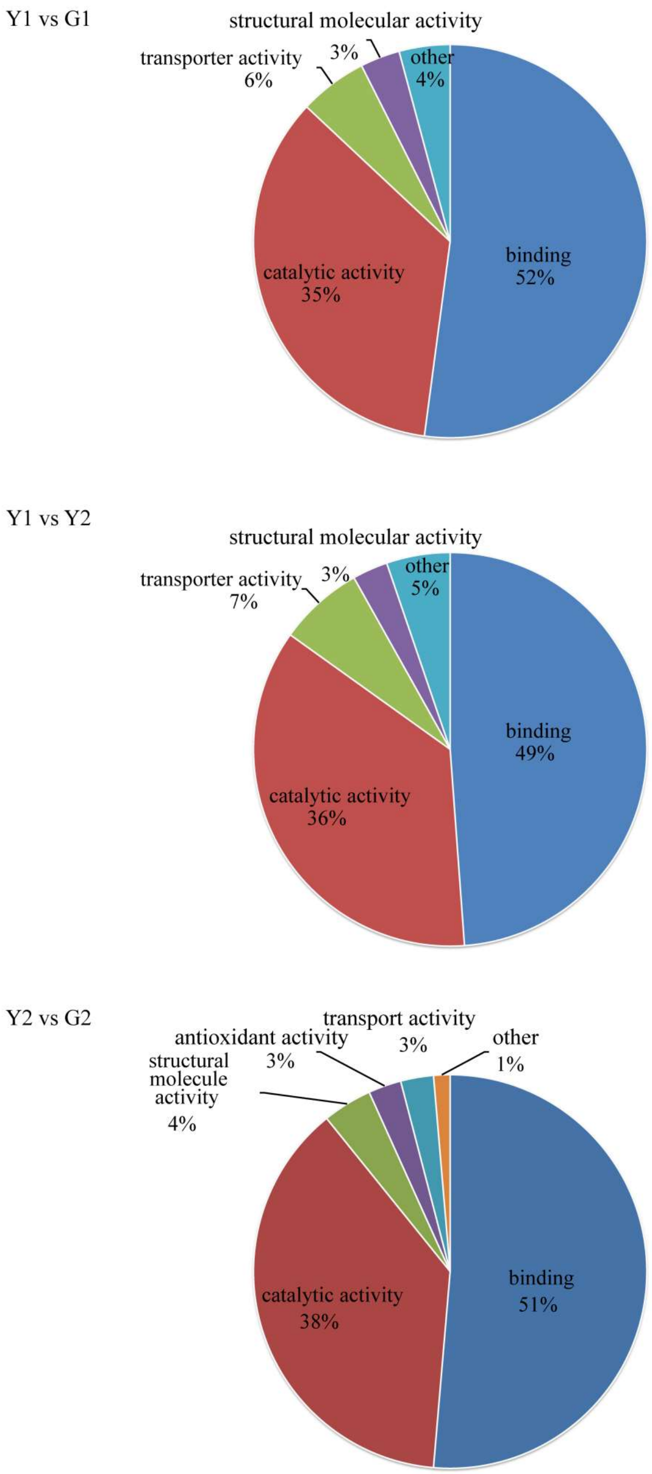

Figure S4. Molecular function of GO function classifications on the phosphorylated proteins in the Y1 vs G1, Y1 vs Y2 and Y2 vs G2 comparisons, respectively.

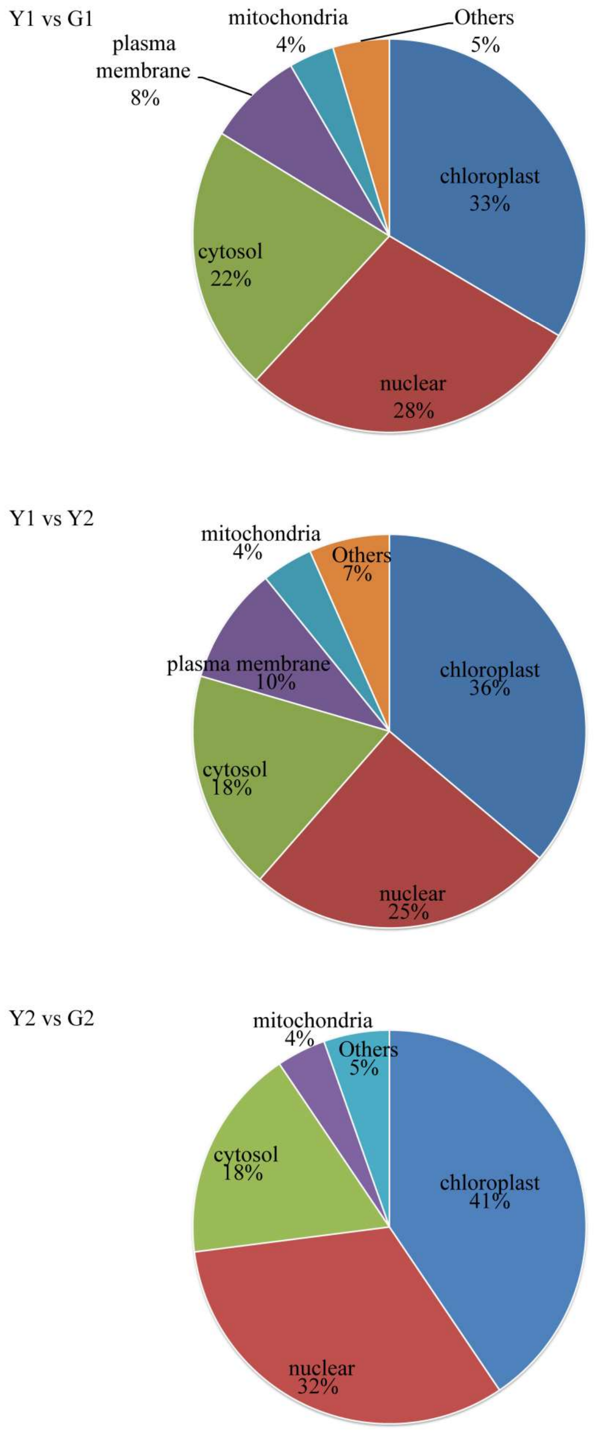

Figure S5. Subcellular localization prediction of GO function classifications on the phosphorylated proteins in the Y1 vs G1, Y1 vs Y2 and Y2 vs G2 comparisons, respectively.

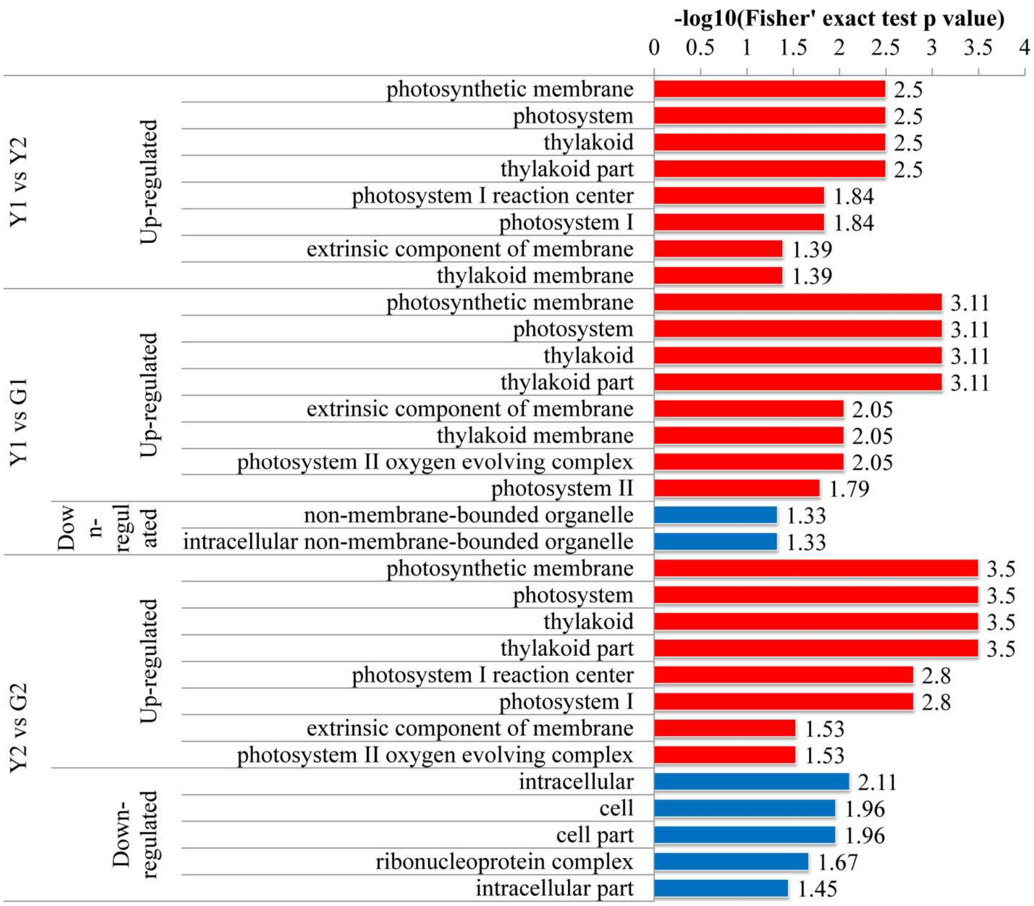

Figure S6. GO enrichment analysis on cellular component in the Y1 vs G1, Y1 vs Y2 and Y2 vs G2 comparisons.

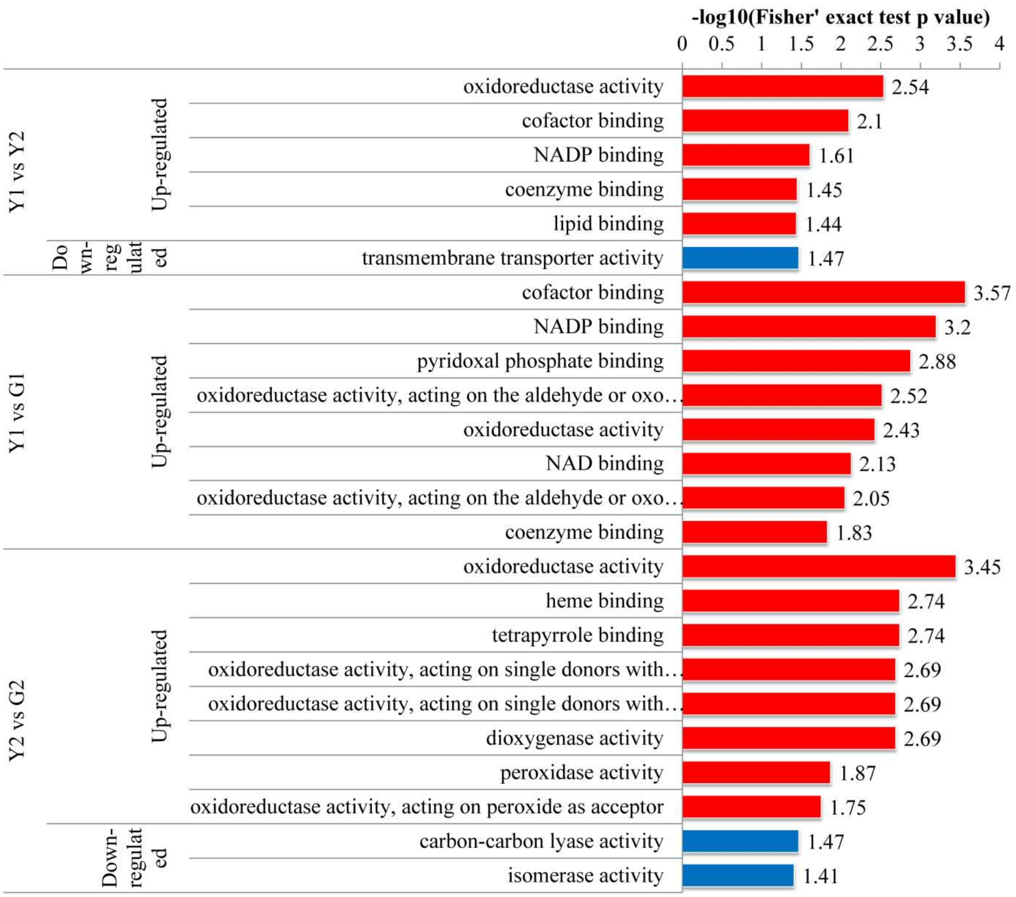

Figure S7. GO enrichment analysis on molecular function in the Y1 vs G1, Y1 vs Y2 and Y2 vs G2 comparisons.

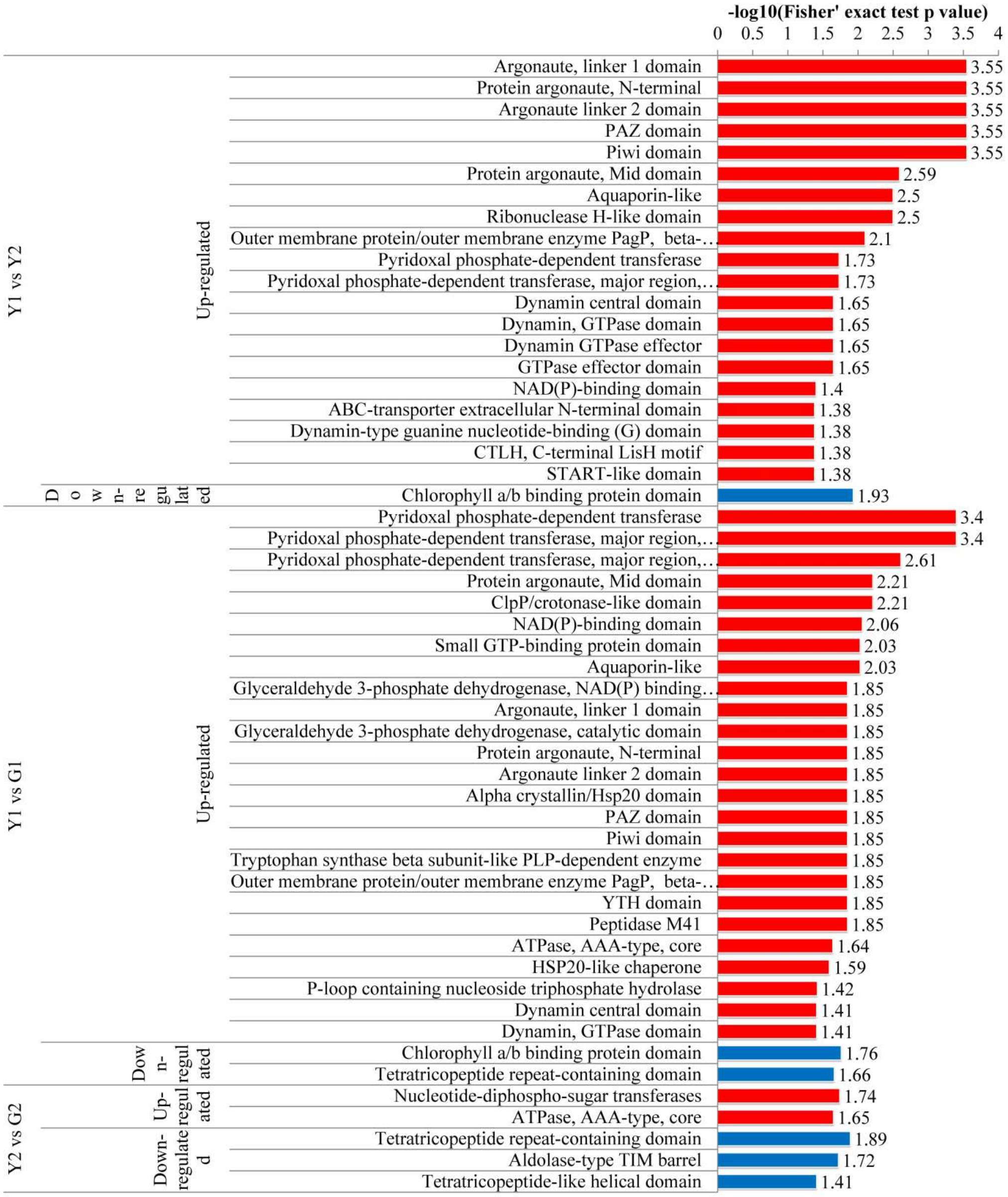

Figure S8. Domain enrichment analyses in the Y1 vs G1, Y1 vs Y2 and Y2 vs G2 comparisons, respectively.

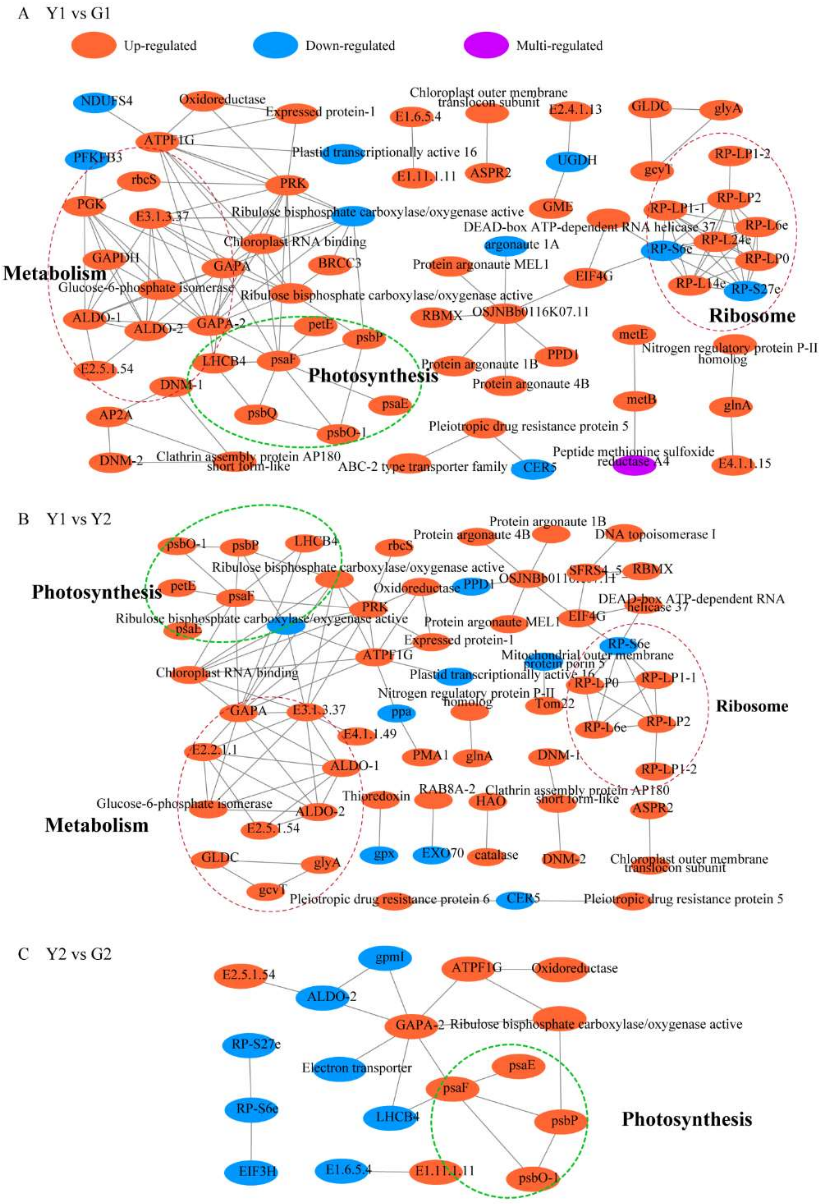

Figure S9. Protein-protein interaction networks of phosphorylated proteins in the Y1 vs G1, Y1 vs Y2 and Y2 vs G2 comparisons.
